# Intermittent Fasting Restores Cardiac Lipid Homeostasis in Diabetic Cardiomyopathy in Association With *Akkermansia Muciniphila* and 1‐methyl‐L‐histidine

**DOI:** 10.1002/advs.76528

**Published:** 2026-07-10

**Authors:** Kaiyuan Jiang, Fen Xiong, Yiwen Peng, Lingfei Meng, Xinran Wang, Yingruo Xu, Tian Tang, Hongchang Gao

**Affiliations:** ^1^ Institute of Metabonomics & Medical NMR School of Pharmaceutical Sciences Wenzhou Medical University Wenzhou China; ^2^ Oujiang Laboratory (Zhejiang Lab For Regenerative Medicine, Vision and Brain Health) Scientific Research Center of Wenzhou Medical University Wenzhou China

**Keywords:** 1‐methyl‐L‐histidine, akkermansia muciniphila, diabetic cardiomyopathy, gut microbiota, intermittent fasting

## Abstract

Diabetic cardiomyopathy (DCM) is a major cardiovascular complication of diabetes with limited effective interventions. Using a streptozotocin‐induced insulin‐deficient, type 1 diabetes‐like DCM mouse model, we show that intermittent fasting (IF) improves cardiac function and attenuates myocardial remodeling. Antibiotic‐mediated microbiota depletion largely abolished these benefits, whereas fecal microbiota transplantation from IF‐treated donors recapitulated cardioprotection, supporting a causal role of the gut microbiota. Metagenomic profiling identified *Akkermansia muciniphila* (*A. muciniphila*) as a prominent IF‐responsive taxon, and *A. muciniphila* supplementation alleviated cardiac injury without obvious improvement in glycaemia. Integrated serum and heart metabolomics identified 1‐methyl‐L‐histidine as a microbiota‐associated metabolite reduced in diabetes but restored by IF and *A. muciniphila*. In vitro and ex vivo assays further supported an L‐anserine‐linked microbial route for 1‐methyl‐L‐histidine generation. Importantly, oral 1‐methyl‐L‐histidine supplementation recapitulated key cardioprotective effects, remodeled cardiac lipid homeostasis, and reduced lipid peroxidation and oxidative injury. Together, these findings support a gut microbiota–metabolite–lipid axis associated with IF‐related cardioprotection in DCM and highlight microbial metabolites as tractable targets to complement dietary intervention.

## Graphical abstract

Intermittent fasting reshapes the gut microbiota in diabetic cardiomyopathy, restoring *Akkermansia muciniphila* and the microbiota‐associated metabolite 1‐methyl‐L‐histidine. This shift is linked to improved cardiac lipid homeostasis, reduced lipid peroxidation, and attenuated myocardial injury, highlighting a gut microbiota–metabolite–lipid axis in diabetic heart disease.

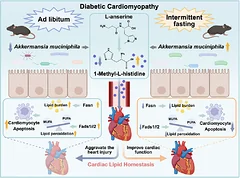

## 1 Introduction

Diabetic cardiomyopathy (DCM) refers to diabetes‐associated myocardial injury that develops in the absence of overt coronary artery disease or hypertension and is characterized by progressive abnormalities in cardiac structure and function [1]. As diabetes becomes increasingly prevalent worldwide, DCM represents an important contributor to heart failure and diabetes‐related mortality. Epidemiological evidence indicates that more than one‐third of patients with diabetes develop DCM, posing a substantial clinical and socioeconomic burden [2, 3]. At the pathological level, DCM is accompanied by a continuum of changes ranging from early relaxation defects to impaired systolic performance, together with cardiomyocyte hypertrophy, mitochondrial dysfunction, impaired metabolic flexibility, oxidative stress, and extracellular matrix remodeling [4]. Although cardiometabolic therapies have improved the management of diabetes and cardiovascular complications, effective strategies specifically targeting DCM remain insufficient, and long‐term adherence to lifestyle interventions is often difficult to maintain [5]. Accordingly, there remains a need for safe and sustainable therapeutic strategies that target the underlying mechanisms of DCM.

DCM is increasingly viewed as a systemic disorder rather than a purely myocardium‐confined consequence of hyperglycaemia. In particular, the gut microbiota has emerged as an important interface between host metabolism, immune regulation, and cardiovascular physiology. Gut dysbiosis has been implicated in heart failure, coronary artery disease, and hypertension through immune activation, microbial metabolite signaling, and host metabolic reprogramming [6, 7, 8, 9]. Meanwhile, both clinical cohorts and diabetic animal models consistently exhibit disrupted gut microbial composition compared with non‐diabetic controls [10, 11]. Increasing evidence further suggests that microbial community alterations and their metabolic products may participate in diabetic cardiac dysfunction, making the gut microbiota a potentially modifiable component of DCM pathogenesis [12]. However, the specific microbial taxa, metabolite signals, and causal gut–heart pathways involved in DCM remain poorly resolved.

Intermittent fasting (IF) imposes recurring fasting–feeding cycles and has emerged as a practical dietary strategy capable of reshaping host metabolic programs [13, 14]. IF improves glycemic control, lipid metabolism, and systemic inflammation, and has shown therapeutic potential in diverse cardiometabolic conditions, including non‐alcoholic fatty liver disease, neurodegeneration, and obesity‐associated cardiomyopathy [15, 16, 17, 18]. Preclinical studies also demonstrate that IF reduces cardiovascular risk factors and improves cardiac bioenergetics and remodeling under metabolic stress [15, 19, 20, 21]. Beyond these host‐centered effects, IF can alter gut microbial community structure and microbial metabolite production, suggesting that intestinal microbial remodeling may contribute to some of its distal organ‐protective actions. Nevertheless, whether IF protects the diabetic heart through a defined microbiota‐dependent metabolite pathway, and which microbial species and downstream metabolites are involved, remains unclear.

Here, using a streptozotocin (STZ)‐induced mouse model of DCM, we examined whether IF attenuates diabetic cardiac injury and asked to what extent this effect depends on the gut microbiota. By combining antibiotic depletion, fecal microbiota transplantation (FMT), microbial profiling, and metabolomics, we identify *Akkermansia muciniphila* (*A. muciniphila*) as a prominent IF‐responsive taxon and 1‐methyl‐L‐histidine as a microbiota‐associated metabolite linked to IF‐related cardioprotection. We further demonstrate that 1‐methyl‐L‐histidine restores membrane lipid homeostasis and suppresses lipid peroxidation injury, thereby attenuating the pathological progression of DCM. Collectively, our findings support a microbiota–metabolite–lipid axis associated with IF‐mediated cardioprotection in diabetic cardiac injury and provide a rationale for exploring microbiome‐informed and metabolite‐based strategies in DCM.

## 2 Results

### 2.1 IF Alleviates Diabetic Cardiomyopathy in STZ‐Induced Mice

To assess whether IF confers cardioprotection in DCM, we established an STZ‐induced type 1 diabetes–like mouse model and applied an alternate‐day 24 h fasting regimen for 8 weeks (Figure 1A). STZ‐treated mice developed sustained hyperglycaemia, which was not significantly improved by IF during the intervention period (Figure 1B). Diabetic mice also exhibited reduced body weight and increased food and water intake relative to controls, whereas IF had little effect on these systemic metabolic parameters (Figure 1C–E). These results indicate that IF did not markedly alter systemic glycaemic status or general metabolic intake/output parameters in diabetic mice.

**FIGURE 1:**
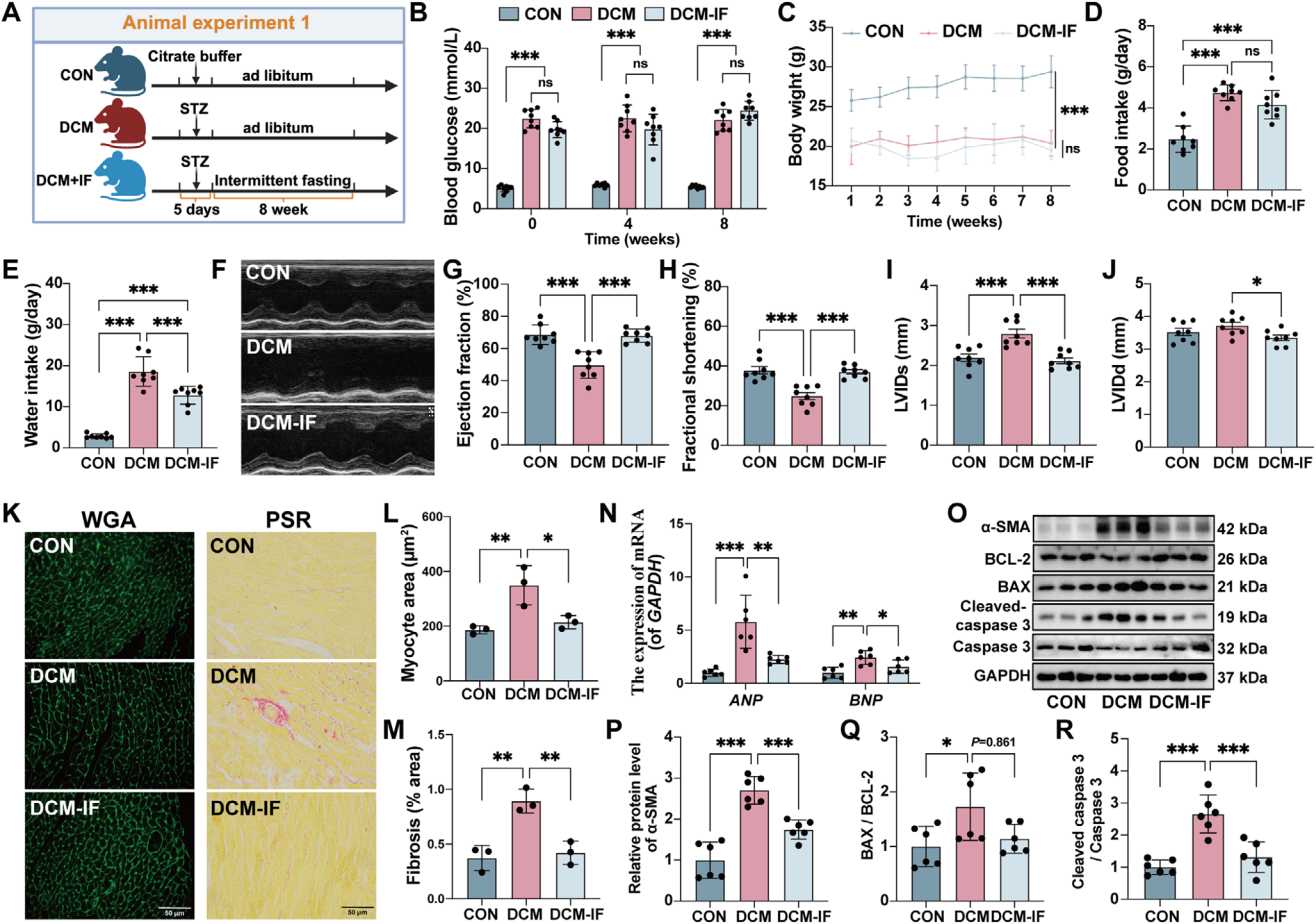
Intermittent fasting alleviates diabetic cardiomyopathy in an STZ‐induced type 1 diabetes‐like mouse model. A. Schematic illustration of animal experiment 1. Male C57BL/6 mice received citrate buffer (CON) or STZ to induce diabetes, followed by ad libitum feeding (DCM) or intermittent fasting (DCM‐IF) for 8 weeks. B. Blood glucose levels at baseline and during intervention (0, 4, and 8 weeks, n = 8 per group). C. Body weight changes during the 8‐week intervention (n = 8 per group). D‐E. Food intake (D) and water intake (E) during the intervention period (n = 8 per group). F. Representative M‐mode echocardiographic images. G‐J. Quantification of echocardiographic parameters, including ejection fraction (EF, G), fractional shortening (FS, H), left ventricular internal dimension at end‐systole (LVIDs, I), and left ventricular internal dimension at end‐diastole (LVIDd, J) (n = 6 per group). K. Representative WGA staining (left) for cardiomyocyte morphology and PSR staining (right) for collagen deposition in cardiac sections. Scale bars, 50 µm. L. Quantification of cardiomyocyte cross‐sectional area (CSA). Each value represents the average CSA of 50 cardiomyocytes measured in one heart section from each mouse (n = 3 per group). M. Quantification of myocardial fibrosis area (n = 3 per group). N. Relative mRNA expression of ANP and BNP in left ventricular tissue (n = 6 per group). O. Representative immunoblots of α‐SMA, BCL‐2, BAX, cleaved‐Caspase‐3, Caspase‐3, and GAPDH in left ventricular tissue. P‐R. Densitometric quantification of α‐SMA, BAX/BCL‐2, and Cleaved‐caspase‐3/Caspase‐3 (n = 6 per group). Data are presented as mean ± SD. One‐way ANOVA followed by Dunnett's multiple comparisons test was used for comparisons among three groups. ^*^ *p* < 0.05, ^**^ *p* < 0.01, ^***^ *p* < 0.001.

We next evaluated cardiac performance by echocardiography. Representative M‐mode images revealed marked systolic dysfunction in DCM mice, whereas IF partially restored contractile activity (Figure 1F). Quantitative analysis showed that DCM mice displayed significantly reduced ejection fraction (EF) and fractional shortening (FS), together with increased left ventricular internal diameter at end‐systole (LVIDs). IF significantly improved EF and FS, accompanied by significant reductions in both LVIDs and left ventricular internal diameter at end‐diastole (LVIDd) (Figure 1G–J). These data demonstrate substantial functional recovery in response to IF. We then examined myocardial remodeling. Wheat germ agglutinin staining showed that IF attenuated diabetes‐associated cardiomyocyte hypertrophy, as reflected by reduced cross‐sectional area (Figure 1K,L). Picrosirius red staining further demonstrated a marked reduction in myocardial fibrosis following IF treatment (Figure 1K–M). Consistently, the cardiac expression of hypertrophic stress markers, including atrial natriuretic peptide (ANP) and brain natriuretic peptide (BNP), was significantly decreased in IF‐treated mice (Figure 1N).

Because cardiomyocyte injury and maladaptive remodeling are central features of DCM progression, we further assessed α‐SMA expression and apoptosis‐related signaling. DCM hearts exhibited increased α‐SMA abundance, an elevated BAX/BCL2 ratio, and increased Cleaved‐caspase‐3/Caspase‐3 ratio, whereas IF markedly reversed these changes (Figure 1O–R and Figure S6A). Together, these results show that IF effectively alleviates systolic dysfunction, structural remodeling, and pro‐apoptotic signaling in STZ‐induced DCM.

### 2.2 IF Remodels the Gut Microbiota in DCM

Given the emerging role of the gut microbiota in cardiometabolic homeostasis, we next asked whether IF alleviates DCM in association with microbial remodeling. Shotgun metagenomic profiling revealed marked alterations in gut microbial composition in diabetic mice relative to controls. At the community level, α‐diversity was reduced in DCM mice, as reflected by a significant decrease in the Shannon index, whereas IF partially restored this loss of diversity (Figure S1A–C). Principal coordinates analysis (PCoA) based on Bray–Curtis distances further showed a clear separation among CON, DCM, and DCM‐IF groups, indicating that IF induced a robust shift in microbial community structure under diabetic conditions (Figure 2A). We next examined taxonomic changes associated with IF. At the phylum level, *Verrucomicrobia* was markedly depleted in DCM mice and substantially restored by IF (Figure 2B,C). To assess whether this lineage contributed prominently to the IF‐associated shift in β‐diversity, we recalculated the PCoA after excluding *Verrucomicrobia*‐associated features. Under this condition, group separation was attenuated and no longer statistically significant, supporting *Verrucomicrobia* as a major contributor to IF‐dependent microbial remodeling (Figure 2D). Consistent with this interpretation, a supplementary genus‐level PERMANOVA analysis incorporating *Akkermansia* abundance as a covariate showed that *Akkermansia* accounted for 9.4% of the variation in community structure, whereas the group effect remained significant after adjustment, further supporting the *Verrucomicrobia*/*Akkermansia* axis as an important contributor to the IF‐associated microbial shift rather than the sole determinant (Table S3). Consistently, genus‐level profiling showed that *Akkermansia* was reduced in DCM mice and enriched following IF (Figure S1D).

**FIGURE 2:**
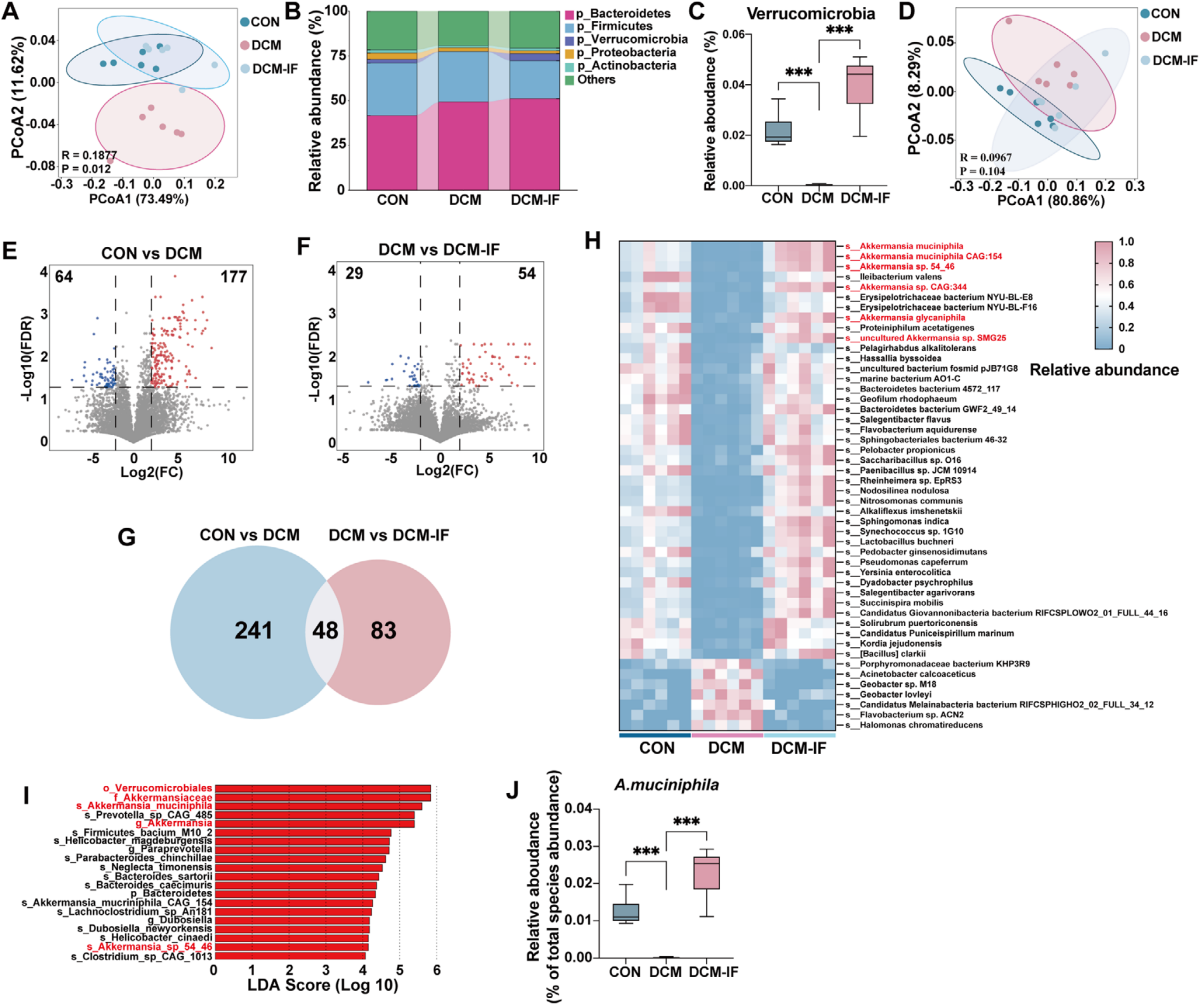
Intermittent fasting remodels gut microbiota composition and restores Akkermansia in diabetic cardiomyopathy. (A) PCoA based on Bray‐Curtis distances showing distinct microbial community structures among CON, DCM, and DCM‐IF groups. PERMANOVA statistics are indicated (n = 6 per group). (B) Relative abundance of dominant bacterial phyla across groups. (C) Relative abundance of the phylum Verrucomicrobia in CON, DCM, and DCM‐IF mice (n = 6 per group). (D) PCoA plot recalculated after excluding Verrucomicrobia‐associated features, indicating that Verrucomicrobia is a major driver of the IF‐associated shift in β‐diversity. PERMANOVA statistics are indicated (n = 6 per group). (E,F) Volcano plots showing differentially abundant taxa between CON vs. DCM (E) and DCM vs. DCM‐IF (F). Taxa were considered significantly differentially abundant when FDR < 0.05 and | log2(FC) | > 2. (G) Venn diagram showing the overlap of differentially abundant taxa identified in (E) and (F). (H) Heatmap of the 48 overlapping differentially abundant species‐level taxa across individual samples. Columns represent samples and rows represent taxa. The color scale represents normalized relative abundance, ranging from low abundance (blue, 0) to high abundance (pink, 1). (I) LEfSe analysis showing taxa with significant differential abundance among groups (LDA score > 4, as indicated). (J) Relative abundance of A. *muciniphila* across groups (species‐level relative abundance, expressed as % of total species abundance; n = 6 per group). Data are presented as box‐and‐whisker plots (median, interquartile range, and min–max) where applicable. For A and D, p values were calculated by PERMANOVA based on Bray–Curtis distances. For C and J, group comparisons were assessed by one‐way ANOVA followed by Dunnett's multiple‐comparisons test, or by nonparametric tests where appropriate. For E‐F, multiple‐testing correction was performed using the Benjamini–Hochberg false discovery rate method. ^*^ *p* < 0.05, ^**^ *p* < 0.01, ^***^ *p* < 0.001.

To systematically identify taxa altered in DCM and reversed by IF, we performed differential abundance analyses using FDR‐controlled criteria, with taxa considered differentially abundant when FDR < 0.05 and |log_2_(fold change, FC)| > 2. Volcano plots revealed broad microbial alterations in DCM relative to controls and a distinct set of taxa shifted by IF (Figure 2E,F). Using the predefined thresholds shown in Figure 2E, F, 289 taxa were differentially abundant in CON vs. DCM, whereas 131 taxa were differentially abundant in DCM vs. DCM‐IF. Venn analysis further identified 48 overlapping species‐level taxa shared between these comparisons, consistent with a subset of microbes altered in diabetes and reversed by IF (Figure 2G). Heatmap visualization of these 48 overlapping species‐level taxa showed that several members of the *Akkermansia* lineage, including *A. muciniphila* and related *Akkermansia* taxa, were depleted in DCM mice and restored after IF (Figure 2H). Consistently, LEfSe analysis identified the *Verrucomicrobia*–*Akkermansiaceae*–*Akkermansia* lineage as a prominent discriminatory feature enriched in CON and DCM‐IF mice relative to DCM mice (Figure 2I and Figure S1F). At the species level, metagenomic abundance analysis further showed that *A. muciniphila* was markedly reduced in DCM mice and significantly restored by IF (Figure 2J), highlighting *A. muciniphila* as a prominent IF‐responsive microbial species. Together, these data show that IF partially restores microbial diversity and robustly remodels gut microbial composition in diabetic mice, with particularly prominent recovery of the *Verrucomicrobia*/*Akkermansia* axis.

### 2.3 Gut Microbiota Mediates the Cardioprotective Effects of IF in DCM

To determine whether the gut microbiota is functionally required for IF‐mediated cardioprotection, we combined fecal microbiota transplantation (FMT) and antibiotic‐mediated microbiota depletion (ABX) in STZ‐induced DCM mice (Figure 3A). Blood glucose levels remained comparably elevated across diabetic groups, including DCM, DCM‐IF, DCM‐IF+ABX, and DCM+FMT mice, indicating that these microbiota‐directed interventions did not substantially alter systemic glycemic status (Figure S2A). To verify the effectiveness of microbiota depletion and transfer in this cohort, we further profiled fecal microbiota after ABX and FMT interventions. α‐diversity analyses and PCoA based on Bray‐Curtis distances showed that antibiotic treatment markedly disrupted microbial richness, diversity, and community structure, whereas DCM+FMT mice exhibited a shift in microbial configuration toward the CON/DCM‐IF groups (Figure S2D–G). Consistently, universal 16S qPCR demonstrated a marked reduction in relative total bacterial load after ABX treatment, confirming effective microbiota depletion, while qPCR analysis showed that *A. muciniphila* abundance was reduced in DCM mice, restored by IF, markedly depleted by ABX, and increased in DCM+FMT mice (Figure S2H,I). With these changes in microbial depletion and transfer confirmed, representative M‐mode images showed that microbiota transfer from IF‐treated donors reproduced the contractile improvement associated with IF, whereas microbiota depletion largely attenuated this benefit (Figure 3B). Quantitative analysis further demonstrated that FMT significantly increased EF and FS, while ABX reduced both parameters toward the DCM phenotype (Figure 3C,D). LVIDs and LVIDd did not differ markedly among CON, DCM, DCM‐IF, DCM‐IF+ABX, and DCM+FMT groups (Figure S2B,C).

**FIGURE 3:**
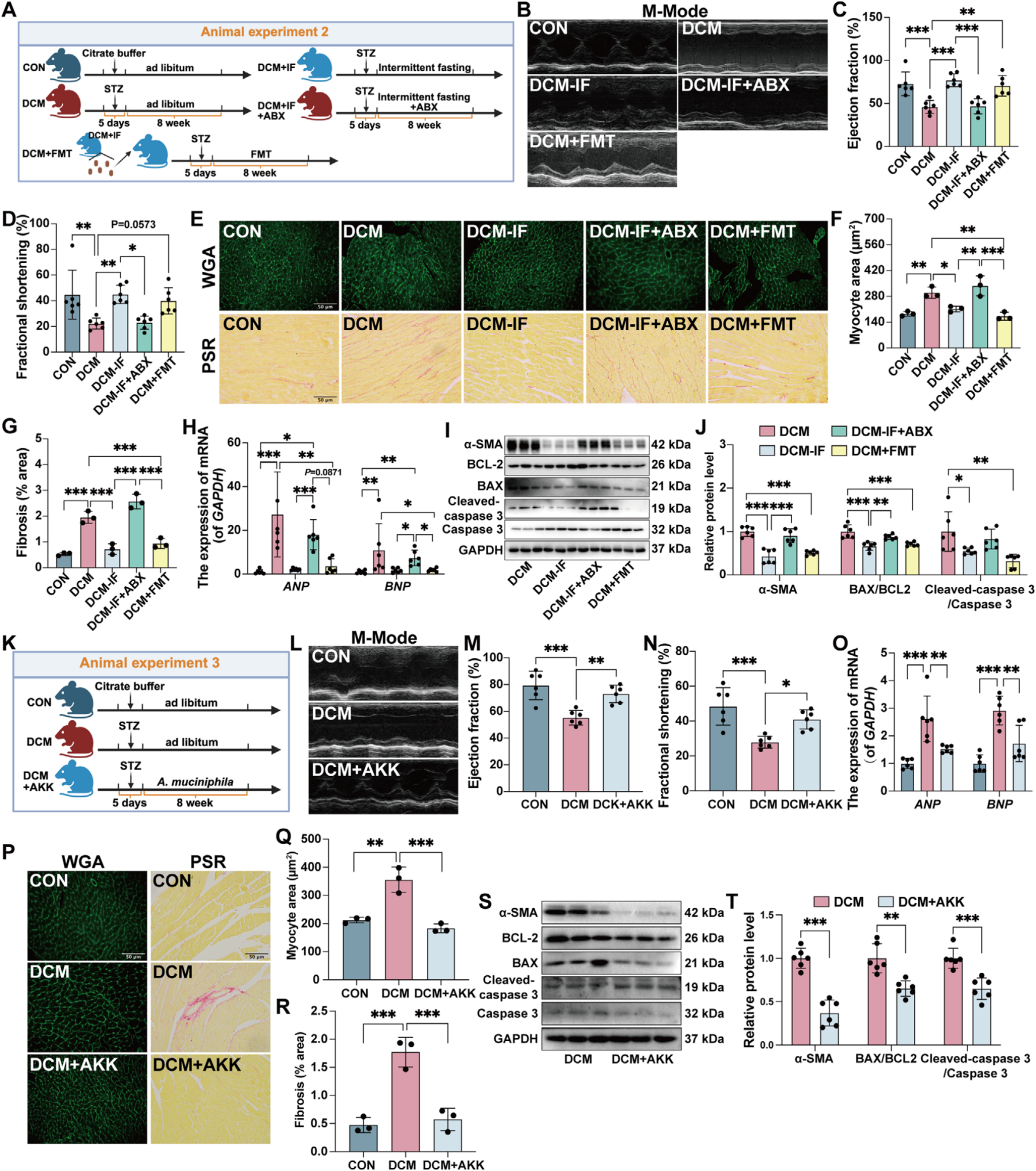
Gut microbiota mediates intermittent fasting‐associated cardioprotection, and *A. muciniphila* supplementation attenuates diabetic cardiomyopathy. (A) Schematic illustration of animal experiment 2, including antibiotic‐mediated microbiota depletion (DCM‐IF+ABX) and fecal microbiota transplantation (DCM+FMT). (B) Representative M‐mode echocardiographic images from CON, DCM, DCM‐IF, DCM‐IF+ABX, and DCM+FMT mice. (C,D) Quantification of ejection fraction (EF, C) and fractional shortening (FS, D) (n = 6 per group). (E) Representative WGA staining (upper) and PSR staining (lower) of myocardial sections from CON, DCM, DCM‐IF, DCM‐IF+ABX, and DCM+FMT mice. Scale bars, 50 µm. (F,G) Quantification of cardiomyocyte cross‐sectional area (F) and myocardial fibrosis area (G) (n = 3 per group). (H) Relative mRNA expression of ANP and BNP in left ventricular tissue (n = 6 per group). (I) Representative immunoblots of α‐SMA, BCL‐2, BAX, cleaved caspase‐3, caspase‐3, and GAPDH in left ventricular tissue from DCM, DCM‐IF, DCM‐IF+ABX, and DCM+FMT mice. (J) Densitometric quantification of α‐SMA, BAX/BCL‐2, and Cleaved caspase‐3/Caspase‐3 (n = 6 per group). (K) Schematic illustration of animal experiment 3. DCM mice received oral supplementation of *A. muciniphila* (DCM+AKK) for 8 weeks. (L) Representative M‐mode echocardiographic images from CON, DCM, and DCM+AKK mice. (M,N) Quantification of EF (M) and FS (N) (n = 6 per group). (O) Relative mRNA expression of ANP and BNP in left ventricular tissue (n = 6 per group). (P) Representative WGA staining (left) and PSR staining (right) of myocardial sections from CON, DCM, and DCM+AKK mice. Scale bars, 50 µm. (Q,R) Quantification of cardiomyocyte cross‐sectional area (Q) and myocardial fibrosis area (R) (n = 3 per group). (S) Representative immunoblots of α‐SMA, BCL‐2, BAX, cleaved caspase‐3, caspase‐3, and GAPDH in left ventricular tissue from DCM and DCM+AKK mice. (T) Densitometric quantification of α‐SMA, BAX/BCL‐2, and Cleaved caspase‐3/Caspase‐3 (n = 6 per group). Data are presented as mean ± SD with individual animals shown as dots. Statistical significance was assessed by one‐way ANOVA followed by Dunnett's multiple‐comparisons test, or by nonparametric tests where appropriate. ^*^ *p* < 0.05, ^**^ *p* < 0.01, ^***^ *p* < 0.001.

Histological analyses were consistent with the functional findings. Representative WGA and PSR staining showed that FMT largely recapitulated the anti‐remodeling effects observed with IF, including attenuation of cardiomyocyte hypertrophy and collagen deposition, whereas ABX largely abolished these IF‐associated improvements (Figure 3E). Quantification confirmed that FMT reproduced the IF‐induced reductions in cardiomyocyte cross‐sectional area and fibrosis area, while microbiota depletion markedly blunted these protective effects of IF (Figure 3F,G). At the molecular level, FMT mirrored the suppressive effect of IF on the hypertrophic stress markers ANP and BNP, whereas ABX reversed this IF‐associated suppression (Figure 3H). Immunoblot analysis further showed that FMT recapitulated the IF‐associated decreases in α‐SMA expression, BAX/BCL2 ratio, and Cleaved‐caspase‐3/Caspase‐3 ratio, while these IF‐induced changes were largely lost after microbiota depletion (Figure 3I,J and Figure S6B). Together, these results indicate that IF‐mediated cardioprotection is microbiota‐dependent, such that IF‐shaped microbiota is sufficient to transfer protection, whereas an intact gut microbiota is required for the full cardioprotective effects of IF.

Given the prominent restoration of *A. muciniphila* following IF, we next tested whether supplementation with *A. muciniphila* could reproduce major IF‐associated benefits in DCM. Blood glucose remained elevated in DCM+AKK mice and was not significantly improved by *A. muciniphila* supplementation (Figure S2J). Despite this, oral administration of *A. muciniphila* improved cardiac performance, as shown by representative M‐mode echocardiography together with increased EF and FS (Figure 3L–N). LVIDs and LVIDd were not markedly altered by *A. muciniphila* treatment (Figure S2K,L). *A. muciniphila* supplementation also attenuated myocardial remodeling, as evidenced by reduced cardiomyocyte hypertrophy and collagen deposition on WGA and PSR staining (Figure 3P), accompanied by decreased cardiomyocyte cross‐sectional area and fibrosis area (Figure 3Q,R). Consistently, *A. muciniphila* lowered the expression of ANP and BNP (Figure 3O) and attenuated α‐SMA expression together with apoptosis‐associated signaling, as reflected by reduced Cleaved‐caspase‐3/Caspase‐3 ratio and a lower BAX/BCL2 ratio (Figure 3S,T and Figure S6C). These findings support that *A. muciniphila* supplementation reproduces major cardioprotective effects associated with IF in diabetic mice, largely independent of overt glycemic correction.

### 2.4 IF Reshapes Systemic and Cardiac Metabolomes and Identifies 1‐Methyl‐L‐Histidine as a Microbiota‐Linked Metabolite Associated With Cardioprotection

Given that metabolites represent a key interface linking the gut microbiota to cardiac function, we next investigated whether IF‐induced metabolic remodeling might underlie microbiota‐dependent cardioprotection in DCM. We therefore performed untargeted metabolomic profiling of serum and heart tissues from CON, DCM, and DCM‐IF mice. Principal component analysis (PCA) revealed distinct metabolic signatures among the three groups in both compartments, with the metabolomes of DCM‐IF mice shifting away from the diabetic state and toward the control state (Figure 4A). A similar separation pattern was observed in negative‐ion mode datasets (Figure S3A), supporting robust IF‐associated metabolic remodeling in both systemic and cardiac compartments.

**FIGURE 4:**
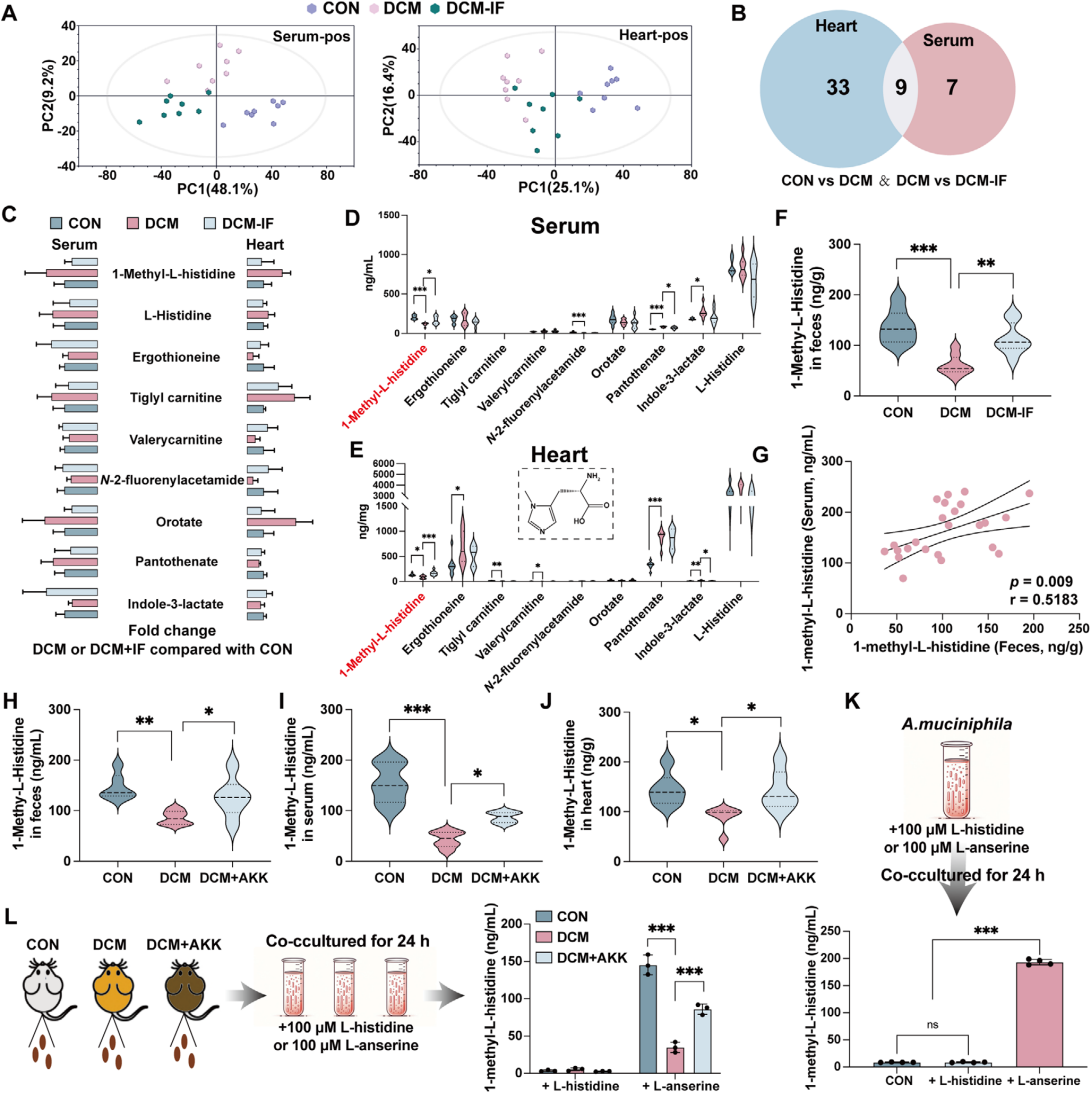
IF‐associated metabolic remodeling identifies 1‐methyl‐L‐histidine as a microbiota‐associated metabolite and associated with *A. muciniphila*. (A) PCA score plots based on untargeted metabolomics (ESI+ mode) showing distinct metabolic profiles in serum (left) and heart tissue (right) among CON, DCM, and DCM‐IF groups (n = 8 per group). (B) Venn diagram illustrating the overlap of FDR‐controlled differential metabolites (FDR < 0.05) that were significantly altered in both comparisons, CON vs. DCM and DCM vs. DCM‐IF, in serum and heart. (C) Fold changes of representative overlapping differential metabolites in serum (left) and heart (right), shown as fold change relative to the CON group. (D,E) Targeted quantification of selected metabolites in serum (D) and heart tissue (E), highlighting 1‐methyl‐L‐histidine (n = 8 per group). (F) Fecal levels of 1‐methyl‐L‐histidine in CON, DCM, and DCM‐IF mice (n = 8 per group). (G) Spearman correlation analysis between fecal and serum 1‐methyl‐L‐histidine levels across individual mice. Spearman's correlation coefficient and p‐value are indicated in the panel. H‐J. Levels of 1‐methyl‐L‐histidine in feces (H), serum (I), and heart tissue (J) in CON, DCM, and DCM+AKK mice (n = 6 per group). (K) In vitro single‐microbial transformation assay showing OD600‐normalized 1‐methyl‐L‐histidine production by A. *muciniphila* after co‐culture with 100 µM L‐histidine or 100 µM L‐anserine for 24 h. (L) Schematic illustration of the in vitro mixed‐microbial transformation assay using fecal microbial communities from CON, DCM, and DCM+AKK mice, followed by co‐culture with 100 µM L‐histidine or 100 µM L‐anserine for 24 h, and quantification of 1‐methyl‐L‐histidine production. Data are presented as mean ± SD or as violin plots with median and quartile lines, as appropriate. For untargeted metabolomic screening in (B,C), multiple‐testing correction was performed using the Benjamini–Hochberg false discovery rate method. Group comparisons were assessed by one‐way ANOVA followed by Dunnett's multiple‐comparisons test or by nonparametric tests where appropriate, and Spearman correlation was used in (G). ^*^ *p* < 0.05, ^**^ *p* < 0.01, ^***^ *p* < 0.001.

We next sought to identify metabolites that were altered in diabetes and reversed by IF in both serum and heart. Differential metabolites were identified using FDR‐controlled criteria based on Benjamini–Hochberg correction within each comparison. By intersecting differential metabolites (FDR < 0.05) shared between the CON vs. DCM and DCM vs. DCM‐IF comparisons across the two compartments, we identified nine common metabolites (Figure 4B), and their relative changes are summarized in Figure 4C. The corresponding untargeted metabolomic statistics, including Log2(FC), raw *P* values, and FDR values, are provided in Tables S4 and S5. We then performed targeted metabolomic analyses to validate these candidate metabolites in serum and heart. Among these, 1‐methyl‐L‐histidine was the only metabolite showing significant and directionally concordant changes in both serum and heart, characterized by reduced levels in DCM mice and restoration after IF intervention (Figure 4D,E). Fecal 1‐methyl‐L‐histidine showed the same decrease‐and‐recovery pattern (Figure 4F). Moreover, fecal and serum 1‐methyl‐L‐histidine levels were positively correlated across individual mice (Figure 4G), supporting concordant intestinal and circulating changes and identifying 1‐methyl‐L‐histidine as a microbiota‐associated metabolite linked to IF‐induced metabolic remodeling.

To further assess whether 1‐methyl‐L‐histidine is linked to microbiota‐dependent remodeling, we examined its abundance in additional intervention paradigms. Antibiotic‐mediated microbiota depletion markedly blunted the IF‐associated increase in 1‐methyl‐L‐histidine across feces, serum, and heart, whereas fecal microbiota transplantation from IF‐treated donors partially recapitulated this restoration (Figure S3B–D). We next asked whether *A. muciniphila*, a prominently restored IF‐responsive taxon, was associated with a similar effect on 1‐methyl‐L‐histidine abundance. In DCM+AKK mice, 1‐methyl‐L‐histidine levels were significantly increased in feces, serum, and heart tissue (Figure 4H–J). Together, these data support 1‐methyl‐L‐histidine as a microbiota‐associated metabolite and suggest that *A. muciniphila* may contribute to its restoration.

We next asked whether 1‐methyl‐L‐histidine could be generated from candidate precursors by microbial communities. In vitro, *A. muciniphila* did not produce detectable 1‐methyl‐L‐histidine from L‐histidine but generated substantial amounts in the presence of L‐anserine (Figure 4K). Importantly, neither L‐histidine nor L‐anserine significantly altered *A. muciniphila* growth, as assessed by OD600 measurements (Figure S3E), indicating that the differential production of 1‐methyl‐L‐histidine was not attributable to changes in bacterial growth. Similarly, mixed microbial communities prepared from fecal samples produced minimal 1‐methyl‐L‐histidine from L‐histidine, whereas L‐anserine supplementation robustly increased 1‐methyl‐L‐histidine production, with the highest levels observed in communities derived from CON and DCM+AKK mice compared with those from DCM mice (Figure 4L). Again, OD600 values were comparable across groups and culture conditions (Figure S3F), supporting that the observed differences reflected altered metabolic conversion rather than growth‐dependent effects. These findings are consistent with L‐anserine serving as a plausible precursor for microbiota‐associated 1‐methyl‐L‐histidine generation and suggest a link between this metabolite and IF‐ and *A. muciniphila*–associated metabolic remodeling.

### 2.5 1‐Methyl‐L‐Histidine Supplementation Ameliorates Diabetic Cardiomyopathy

We next examined whether direct supplementation of 1‐methyl‐L‐histidine could reproduce cardioprotective effects in vivo. In an independent intervention experiment, STZ‐induced DCM mice received oral administration of 1‐methyl‐L‐histidine for 8 weeks (Figure 5A). Targeted analysis showed that 1‐methyl‐L‐histidine supplementation significantly increased 1‐methyl‐L‐histidine levels in serum, heart tissue, and feces in DCM+His mice compared with untreated DCM controls, confirming effective systemic and intestinal exposure (Figure 5B,C and Figure S4A). Notably, blood glucose remained elevated and was not significantly altered by 1‐methyl‐L‐histidine administration (Figure S4B), indicating that 1‐methyl‐L‐histidine supplementation did not markedly affect systemic glycemic status in diabetic mice.

**FIGURE 5:**
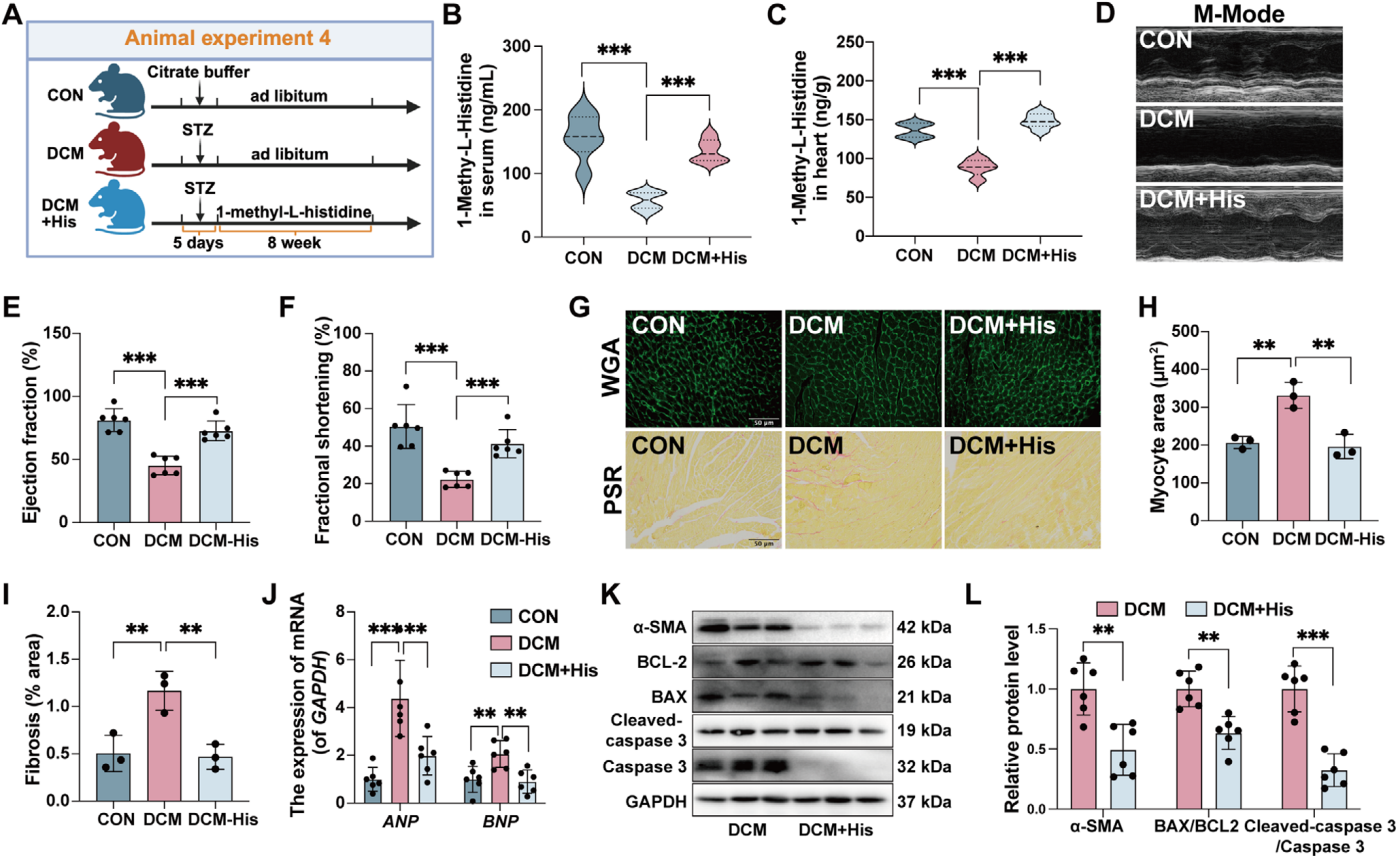
*1‐methyl‐L‐histidine supplementation ameliorates diabetic cardiomyopathy*. (A) Schematic illustration of animal experiment 4. DCM mice received oral administration of 1‐methyl‐L‐histidine (DCM+His) for 8 weeks. (B,C) Levels of 1‐methyl‐L‐histidine in serum (B) and heart tissue (C) from CON, DCM, and DCM+His mice (n = 6 per group). (D) Representative M‐mode echocardiographic images from CON, DCM, and DCM+His mice. (E,F) Quantification of EF (E) and FS (F) (n = 6 per group). (G) Representative WGA staining (upper) and PSR staining (lower) of myocardial sections from CON, DCM, and DCM+His mice. Scale bars, 50 µm. (H,I) Quantification of cardiomyocyte cross‐sectional area (H) and myocardial fibrosis area (I) (n = 3 per group). (J) Relative mRNA expression of ANP and BNP in left ventricular tissue (n = 6 per group). (K) Representative immunoblots of α‐SMA, BCL‐2, BAX, cleaved caspase‐3, caspase‐3, and GAPDH in left ventricular tissue from DCM and DCM+His mice. (L) Densitometric quantification of α‐SMA, BAX/BCL‐2, and Cleaved caspase‐3/Caspase‐3 (n = 6 per group). Data are presented as mean ± SD with individual animals shown as dots. Statistical significance was assessed by one‐way ANOVA followed by Dunnett's multiple‐comparisons test, or by nonparametric tests where appropriate. ^*^ *p* < 0.05, ^**^ *p* < 0.01, ^***^ *p* < 0.001.

We next evaluated cardiac function. Representative M‐mode echocardiographic images showed improved contractile activity in DCM+His mice relative to untreated DCM mice (Figure 5D). Quantitative analysis further demonstrated that 1‐methyl‐L‐histidine significantly increased EF and FS (Figure 5E,F). In addition, LVIDs was significantly reduced, whereas LVIDd showed a partial improvement trend (Figure S4C,D). These findings indicate that 1‐methyl‐L‐histidine supplementation alleviates systolic dysfunction in diabetic hearts. Histological analyses were consistent with these functional improvements. Representative WGA and PSR staining showed that 1‐methyl‐L‐histidine supplementation attenuated cardiomyocyte hypertrophy and collagen deposition in DCM hearts (Figure 5G). Quantification confirmed significant reductions in cardiomyocyte cross‐sectional area and fibrosis area (Figure 5H,I). At the molecular level, 1‐methyl‐L‐histidine reduced the expression of the hypertrophic stress markers ANP and BNP (Figure 5J). Immunoblot analysis further showed that 1‐methyl‐L‐histidine decreased α‐SMA abundance and attenuated pro‐apoptotic signaling, as reflected by a reduced BAX/BCL2 ratio and a lower Cleaved‐caspase‐3/Caspase‐3 ratio (Figure 5K,L and Figure S6D). Together, these data support that direct 1‐methyl‐L‐histidine supplementation reproduces major cardioprotective effects associated with IF and *A. muciniphila* in diabetic mice.

### 2.6 1‐Methyl‐L‐Histidine Remodels Cardiac Lipid Homeostasis and Attenuates Lipid Peroxidation in Diabetic Hearts

Given that dysregulated cardiac lipid metabolism and lipotoxicity are central pathogenic features of diabetic cardiomyopathy [22, 23], we performed comprehensive lipidomic profiling of cardiac tissues from CON, DCM, and DCM+His mice. PCA revealed a clear separation among groups, and the lipidomic profile of DCM+His hearts partially shifted toward that of controls (Figure 6A), indicating broad remodeling of the cardiac lipid landscape following 1‐methyl‐L‐histidine supplementation.

**FIGURE 6:**
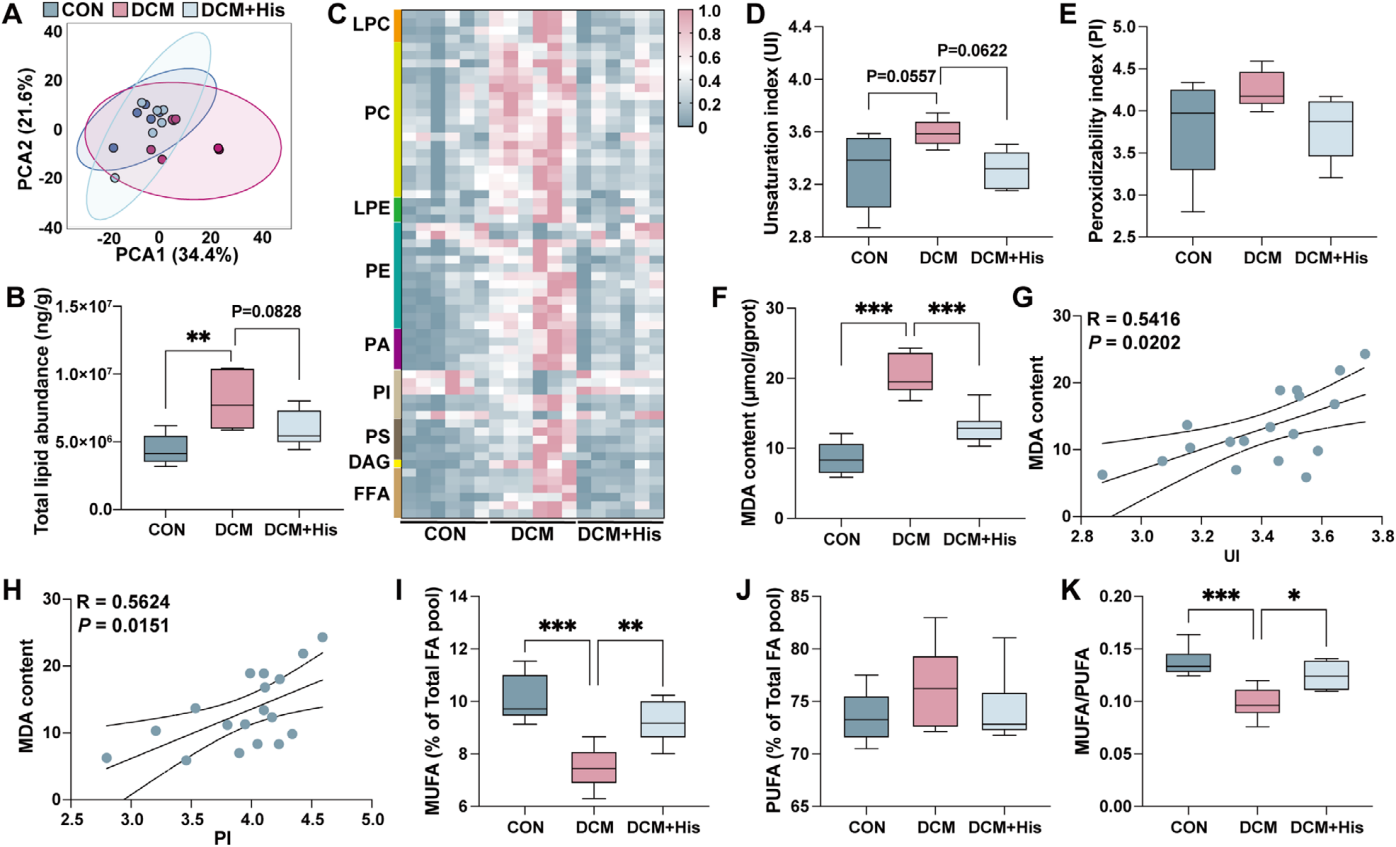
1‐methyl‐L‐histidine restores cardiac lipid homeostasis in diabetic cardiomyopathy. (A) PCA of cardiac lipidomic profiles showing distinct clustering among CON, DCM, and 1‐methyl‐L‐histidine–treated DCM (DCM+His) groups (n = 6 per group). (B) Total cardiac lipid abundance quantified from lipidomic data (n = 6 per group, normalized to tissue weight, ng/g). (C) Heatmap of the overlapping differentially abundant lipids identified in both comparisons, CON vs. DCM and DCM vs. DCM+His (p < 0.05), with lipid species annotated by class. (D) Unsaturation index (UI) calculated as the abundance‐weighted mean number of double bonds across lipid species in each sample (n = 6 per group). (E) Peroxidizability index (PI) calculated using established unsaturation‐class coefficients to estimate peroxidation susceptibility (n = 6 per group). (F) Myocardial malondialdehyde (MDA) content (normalized to protein) as a readout of lipid peroxidation (n = 6 per group). (G,H) Pearson correlation analyses between lipid peroxidation and lipid unsaturation metrics showing the associations of MDA content with UI (G) and PI (H) (n = 6 per group). (I,J) Relative contributions of MUFA (I) and PUFA (J) within the membrane phospholipid fatty‐acid pool. MUFA/PUFA were calculated after excluding sphingomyelin (SM), ceramides (Cer), triacylglycerols (TAG), and other non‐membrane lipid classes (n = 6 per group). (K) MUFA‐to‐PUFA ratio within the membrane phospholipid fatty‐acid pool (n = 6 per group). Data are presented as box‐and‐whisker plots (median, interquartile range, and min–max) unless otherwise indicated. For B, D‐F, and I‐K, group comparisons were assessed by one‐way ANOVA followed by Dunnett's multiple‐comparisons test, or by nonparametric tests where appropriate. For G‐H, Pearson correlation coefficients (R) and two‐tailed p values are provided in (G,H). ^*^ *p* < 0.05, ^**^ *p* < 0.01, ^***^ *p* < 0.001.

Consistent with this global shift, total cardiac lipid abundance was elevated in DCM hearts and was reduced after 1‐methyl‐L‐histidine treatment (Figure 6B). Differential lipid analysis further identified an overlapping set of lipids altered in both CON vs. DCM and DCM vs. DCM+His comparisons (*P*<0.05), which were predominantly enriched in membrane phospholipid classes (Figure 6C), suggesting that membrane lipid remodeling represents a major component of the diabetic lipid phenotype and its reversal by 1‐methyl‐L‐histidine. To quantify compositional features linked to oxidation susceptibility, we calculated the unsaturation index (UI) and peroxidizability index (PI). Both indices trended upward in DCM hearts and downward after 1‐methyl‐L‐histidine supplementation (Figure 6D,E). Importantly, biochemical measurement of malondialdehyde (MDA) demonstrated a marked increase in lipid peroxidation in DCM hearts that was significantly attenuated by 1‐methyl‐L‐histidine (Figure 6F). Across individual animals, MDA levels correlated positively with unsaturation Index (UI) and peroxidizability Index (PI) (Figure 6G,H), supporting a close link between lipid compositional remodeling and oxidative damage. Analysis of fatty‐acid composition within the membrane phospholipid pool further showed reduced monounsaturated fatty acid (MUFA) abundance, a relative shift toward polyunsaturated fatty acid (PUFA) enrichment, and a decreased MUFA/PUFA ratio in DCM hearts, all of which were partially normalized by 1‐methyl‐L‐histidine (Figure 6I–K), consistent with a shift toward a more oxidation‐resistant membrane lipid profile.

To link these lipidomic changes to candidate regulatory nodes, we examined transcripts encoding enzymes governing fatty‐acid synthesis, remodeling, and glycerophospholipid biosynthesis (Figure 7A,B). Among the genes surveyed, *Fasn*, *Fads1*, *Fads2*, and *Gpat2* emerged as candidate nodes showing consistent DCM‐associated changes and reversal by 1‐methyl‐L‐histidine, which were further validated by qPCR (Figure 7C–F). Specifically, *Fasn* was upregulated in DCM hearts and suppressed by 1‐methyl‐L‐histidine, aligning with the observed reduction in total lipid burden (Figures 6B and 7C). *Fads1* and *Fads2* were also induced in DCM and normalized by 1‐methyl‐L‐histidine treatment (Figure 7D,E), consistent with altered fatty‐acid remodeling accompanying the compositional shift in membrane lipids (Figure 6I–K). In addition, *Gpat2*, an upstream entry enzyme for glycerolipid and glycerophospholipid synthesis via LPA formation, was markedly increased in DCM hearts and decreased following 1‐methyl‐L‐histidine supplementation (Figure 7F), providing a transcriptional correlate for the predominant phospholipid perturbations identified in lipidomics (Figure 6C).

**FIGURE 7:**
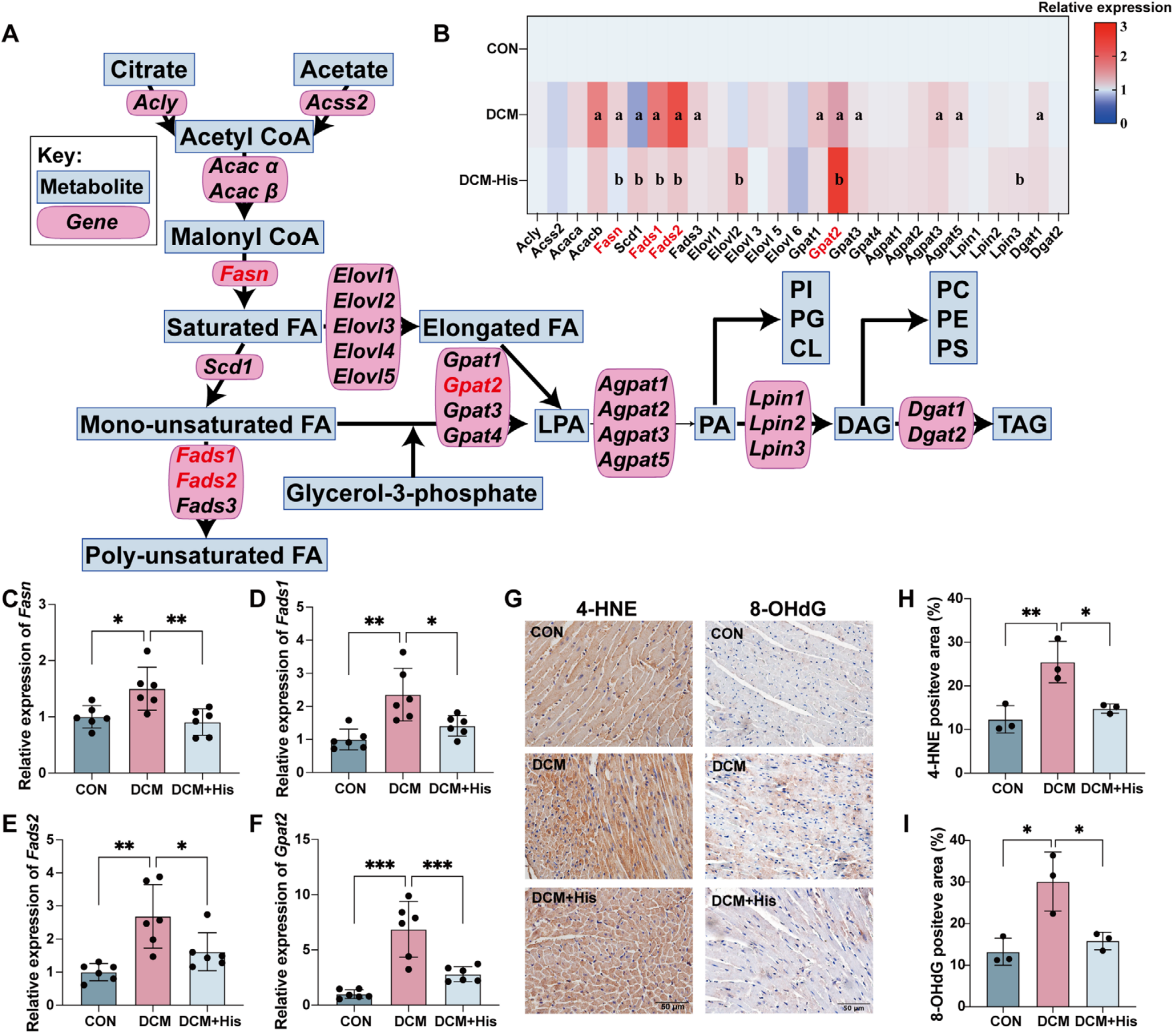
Transcriptional remodeling of lipid metabolic pathways and validation of oxidative injury in diabetic hearts following 1‐methyl‐L‐histidine treatment. (A) Schematic overview of key pathways involved in de novo lipogenesis, fatty‐acid desaturation/elongation, and glycerolipid/glycerophospholipid synthesis, with metabolite nodes and corresponding regulatory genes highlighted. (B) Heatmap summarizing relative mRNA expression changes of lipid metabolism–related genes in left ventricular tissue from CON, DCM, and DCM+His groups. The color scale indicates relative expression levels from low (blue) to high (red). Letters indicate significant pairwise differences, where a denotes DCM vs. CON and b denotes DCM vs. DCM+His. Genes highlighted in red represent those showing consistent changes across comparisons (n = 6 per group). (C–F) qPCR validation of key differentially expressed genes in left ventricular tissue, including Fasn (C), Fads1 (D), Fads2 (E), and Gpat2 (F) (n = 6 per group). (G) Representative immunohistochemical staining of 4‐hydroxynonenal (4‐HNE) and 8‐hydroxy‐2’‐deoxyguanosine (8‐OHdG) in myocardial sections from CON, DCM, and DCM+His mice. Scale bars, 50 µm. (H,I) Quantification of 4‐HNE–positive area (H) and 8‐OHdG–positive area (I) (n = 3 per group). Data are presented as mean ± SD with individual animals shown as dots. Statistical significance was assessed by one‐way ANOVA followed by Dunnett's multiple‐comparisons test, or by nonparametric tests where appropriate. ^*^ *p* < 0.05, ^**^ *p* < 0.01, ^***^ *p* < 0.001.

Finally, we assessed tissue‐level oxidative injury. Immunohistochemical staining revealed increased accumulation of the lipid peroxidation adduct 4‐HNE and the oxidative DNA damage marker 8‐OHdG in diabetic hearts, both of which were significantly reduced by 1‐methyl‐L‐histidine (Figure 7G–I). Cardiac 1‐methyl‐L‐histidine levels were inversely correlated with UI and PI (Figure S5A,B), further supporting an association between this metabolite and a less peroxidation‐prone lipid state. Consistent reductions in 4‐HNE and 8‐OHdG were also observed across IF‐based and microbiota‐dependent intervention paradigms (Figure S5C–E). Collectively, these results indicate that 1‐methyl‐L‐histidine alleviates diabetic cardiac injury by reducing lipid burden, remodeling membrane lipid composition, and limiting lipid peroxidation and oxidative damage in the diabetic heart.

## 3 Discussion and Conclusion

This study outlines a gut microbiota‐dependent mechanism through which IF is associated with cardiac protection in DCM. By integrating metagenomic profiling, metabolomic analyses, microbiota transfer and depletion strategies, single‐species supplementation, and metabolite intervention, we identified *A. muciniphila* as a prominent IF‐responsive taxon and 1‐methyl‐L‐histidine as a microbiota‐associated metabolite that is reduced under diabetic conditions but restored by IF and *A. muciniphila* supplementation. Supplementing 1‐methyl‐L‐histidine directly reproduced key protective phenotypes, including improved cardiac performance, reduced pathological remodeling, and attenuation of oxidative injury. Mechanistically, these protective effects were accompanied by broad remodeling of cardiac lipid homeostasis, reduced lipid peroxidation, and a shift toward a less oxidation‐prone membrane lipid state. Together, our data support an association between microbial metabolic remodeling, cardiac lipid reprogramming, and oxidative injury in IF‐associated cardioprotection.

The STZ‐induced model used here primarily reflects insulin‐deficient, type 1 diabetes–like cardiomyopathy and is therefore well suited for interrogating gut–heart mechanisms in a hyperglycemic setting [24, 25]. Accordingly, our findings should be interpreted primarily within the context of insulin‐deficient diabetic cardiomyopathy, and further validation in type 2 diabetes or insulin resistance models will be required to determine broader applicability. IF is increasingly recognized as a non‐pharmacological intervention with benefits across metabolic and cardiovascular disorders [15, 21, 26]. In mice, alternate‐day 24‐h fasting provides a controllable paradigm for studying repeated fasting–feeding transitions without altering dietary composition and has been associated with broad metabolic and organ‐protective benefits across disease contexts [27, 28]. Beyond systemic metabolic effects, IF can reshape gut microbial ecology and microbial metabolism, thereby influencing host metabolic programs in distal organs [16, 29, 30]. This is particularly relevant to diabetic complications, where dysbiosis and altered microbial metabolites have been linked to chronic inflammation [31, 32], barrier dysfunction [33, 34], and metabolic disturbances [35, 36]. In DCM, the mechanistic basis for IF‐mediated cardioprotection remains incompletely understood. Our ABX and FMT experiments add functional evidence to this concept by showing that the microbiota is not merely associated with IF‐induced protection but contributes to the transfer and maintenance of the protective cardiac phenotype.

Among IF‐responsive microbial changes, the *Verrucomicrobia*/*Akkermansia* lineage emerged consistently across compositional analyses, with *A. muciniphila* robustly restored by IF. Notably, reduced *Akkermansia* abundance has been repeatedly reported in diabetes and related metabolic disorders, and our data are consistent with these observations by showing a marked depletion of *A. muciniphila* in DCM [37, 38, 39]. *A. muciniphila* has attracted attention as a next‐generation probiotic candidate due to its links to metabolic health, gut barrier integrity, and host immune‐metabolic regulation [40]. Preclinical studies have shown that, in some experimental settings, live or pasteurized *A. muciniphila*, as well as selected bacterial components or extracellular vesicles, can improve metabolic dysfunction, intestinal barrier impairment, inflammation, and tissue injury in experimental models [41, 42, 43]. Importantly, clinical translational evidence from a proof‐of‐concept human trial showed that pasteurized *A. muciniphila* was safe and well tolerated in overweight or obese insulin‐resistant volunteers and improved several metabolic parameters [44]. In cardiovascular and cardiometabolic contexts, *A. muciniphila* has been reported to modulate TMAO‐induced cardiac pyroptosis in cold‐related atrial fibrillation and to secrete a GLP‐1‐inducing protein that improves glucose homeostasis [45, 46]. Consistent with this literature, *A. muciniphila* supplementation in our study improved cardiac function and attenuated remodeling in diabetic mice. These findings extend previous metabolic and cardiovascular observations by linking IF‐associated *A. muciniphila* restoration to 1‐methyl‐L‐histidine recovery, reduced lipid peroxidation, and diminished oxidative damage in the diabetic myocardium. Nevertheless, the biological effects of *A. muciniphila* should not be interpreted as universally beneficial. Critical perspectives have emphasized that *A. muciniphila* supplementation may be context‐dependent and should be carefully evaluated in intestinal inflammatory, infectious, or neurologic disease settings [47]. In addition, studies in multiple sclerosis and experimental autoimmune encephalomyelitis suggest that increased *A. muciniphila* abundance or colonization may have divergent effects depending on the surrounding microbial ecosystem and disease context [48]. Therefore, while our findings support a potential cardioprotective association of IF‐associated *A. muciniphila* restoration in insulin‐deficient DCM, they should be viewed within a broader community context rather than as evidence for a single‐taxon mechanism. The translational relevance of *A. muciniphila*‐based strategies requires further validation.

Metabolites provide a direct chemical route through which intestinal microbes can influence extraintestinal tissues. Here, metabolomic screening in IF‐treated mice identified 1‐methyl‐L‐histidine as a candidate metabolite associated with cardioprotection. Its microbiota dependence was supported by antibiotic depletion and fecal microbiota transplantation and further reinforced by the restoration of 1‐methyl‐L‐histidine in *A. muciniphila*‐treated mice. Our in vitro and ex vivo data are consistent with an anserine‐linked microbial route for 1‐methyl‐L‐histidine generation, whereas L‐histidine did not appear to serve as an efficient precursor under our experimental conditions. However, these assays do not definitively establish the in vivo microbial origin of 1‐methyl‐L‐histidine or identify the responsible enzymatic machinery. Although 1‐methyl‐L‐histidine has previously been reported mainly as a biomarker related to meat intake, protein turnover, and disease‐associated metabolic alterations, its functional relevance in microbiota–host communication has remained largely unexplored [49, 50, 51]. Our findings suggest that 1‐methyl‐L‐histidine may function not only as a metabolic marker but also as a candidate microbial‐linked effector associated with diabetic cardiac protection.

Cardiac lipid imbalance is a central metabolic feature of DCM and can promote contractile dysfunction, remodeling, and oxidative injury [22, 23]. In this study, lipidomic profiling indicated that diabetic hearts exhibit broad lipid perturbations with increased total lipid burden and prominent enrichment of differentially regulated species within membrane phospholipid classes. These alterations were partially reversed by 1‐methyl‐L‐histidine supplementation, suggesting that this metabolite engages lipid homeostatic programs in the diabetic myocardium. The functional relevance of this lipid remodeling was supported by reduced MDA accumulation and lower myocardial 4‐HNE and 8‐OHdG staining after 1‐methyl‐L‐histidine treatment. Notably, lipid unsaturation metrics were coupled to oxidative damage at the individual level, and cardiac 1‐methyl‐L‐histidine levels were inversely associated with UI and PI, supporting a link between a less peroxidation‐prone lipid state and reduced oxidative injury.

At the transcriptional level, several lipid metabolic nodes emerged as consistent correlates of the lipidomic phenotype. *Fasn* was induced in diabetic hearts and suppressed by 1‐methyl‐L‐histidine, aligning with the reduction in total lipid burden and supporting the relevance of targeting lipogenic programs in cardiometabolic injury. Prior work has shown that manipulating lipid‐driven lipotoxic pathways, including ceramide‐related programs, can attenuate lipotoxic cardiomyopathy [52]. In addition, our data highlight remodeling of fatty acid composition within the membrane phospholipid pool, characterized by reduced MUFA abundance and a lower MUFA/PUFA ratio in DCM that was restored after 1‐methyl‐L‐histidine treatment, a pattern consistent with improved resistance to oxidative damage. This compositional remodeling was accompanied by normalization of *Fads1/2* expression, suggesting that fatty acid remodeling and PUFA‐related metabolic wiring may be altered in the diabetic heart. While evidence connecting FADS1/2 to cardiac phenotypes remains limited and heterogeneous, genetic and biomarker studies have implicated the FADS locus in cardiovascular conditions [53]. Moreover, *Gpat2*, an entry enzyme for glycerolipid and glycerophospholipid synthesis through LPA formation, was markedly increased in DCM hearts and reduced by 1‐methyl‐L‐histidine, providing a plausible transcriptional correlate for the predominant membrane phospholipid disturbances observed in lipidomics. Together, these observations suggest that 1‐methyl‐L‐histidine does not simply suppress oxidative injury downstream, but is associated with a broader shift in lipid synthesis, remodeling, and membrane susceptibility to peroxidation.

Taken together, these findings support IF as a potentially tractable dietary strategy for alleviating diabetic cardiomyopathy and highlight gut–heart crosstalk as a mechanistically actionable layer of cardiometabolic regulation. By connecting IF‐induced microbial remodeling, 1‐methyl‐L‐histidine restoration, cardiac lipid reprogramming, and oxidative injury, this study provides an integrated view of how dietary intervention may engage the gut microbiota to influence the diabetic heart. These results further suggest that microbiome‐informed and metabolite‐based strategies may complement lifestyle intervention for DCM, while further studies in type 2 diabetes or insulin resistance models are needed to establish broader translational relevance.

## 4 Limitations

Several limitations should be acknowledged. First, this study was performed in an STZ‐induced insulin‐deficient, type 1 diabetes‐like DCM model. Although this model is well suited for studying hyperglycemia‐associated cardiac injury, it does not fully capture the metabolic complexity of type 2 diabetes or insulin resistance‐associated DCM. Future studies in type 2 diabetes models and clinical cohorts are needed to determine the broader translational relevance of the IF–microbiota–1‐methyl‐L‐histidine axis. Second, while antibiotic depletion, fecal microbiota transplantation, and *A. muciniphila* supplementation support a microbiota‐dependent contribution to cardioprotection, the gut microbial community changes induced by IF are likely broader than a single taxon or metabolite, and the contribution of additional IF‐responsive microbial functions remains to be defined. Moreover, although *A. muciniphila* and 1‐methyl‐L‐histidine were associated with major IF‐related protective effects in the present study, we did not perform loss‐of‐function or rescue experiments to establish whether either is necessary for IF‐mediated cardioprotection. Third, although our lipidomic, transcriptional, and intervention data support a link between 1‐methyl‐L‐histidine and cardiac lipid remodeling, we did not directly test the causal roles of Fasn, Gpat2, Fads1, or Fads2, nor did we identify the specific microbial enzymes and genetic determinants responsible for the conversion of L‐anserine to 1‐methyl‐L‐histidine. These mechanistic questions warrant further investigation.

## 5 Experimental Section

### 5.1 Ethics Statement

All animal procedures were performed in accordance with the Guide for the Care and Use of Laboratory Animals and were approved by the Ethics Committee of Wenzhou Medical University (No. xmsq2024‐0703).

### 5.2 Animals and Experimental Design

Specific‐pathogen‐free male C57BL/6J mice (7 weeks old) were purchased from Vital River Laboratory Animal Technology Co., Ltd. (Zhejiang, China) and housed under a 12‐h light/dark cycle with ad libitum access to water and standard chow unless otherwise specified. Mice were randomly assigned into experimental groups as described below. For each cohort, cardiac tissues were prospectively allocated to different downstream applications at the time of tissue collection. In three mice per group, half of the heart was fixed and paraffin‐embedded for histological and immunohistochemical analyses, while the remaining half was reserved for biochemical assays. The other three hearts per group were used for molecular and biochemical analyses, including western blotting, RNA analysis, metabolomics, and lipidomics. Therefore, differences in sample size across assays reflected planned tissue allocation, and no animals were excluded after data acquisition.

### 5.3 Blood and Tissue Collection

At the experimental endpoint, mice were first subjected to echocardiographic assessment and then anesthetized with chloral hydrate for terminal sample collection. Blood was collected by orbital exsanguination and allowed to clot at room temperature for 2 h. Serum was obtained by centrifugation at 4000 rpm for 15 min at 4°C, and the supernatant was collected and stored at −80°C until analysis. After blood collection, mice were euthanized by cervical dislocation. Hearts were immediately excised, rinsed in cold PBS, and processed according to downstream applications. For molecular and metabolomic analyses, left ventricular tissues were dissected, snap‐frozen in liquid nitrogen, and stored at −80°C. For histological analyses, hearts were fixed in 4% paraformaldehyde and paraffin‐embedded for sectioning.

### 5.4 Diabetic Cardiomyopathy Model

A streptozotocin (STZ)‐induced type 1 diabetes (T1D)‐like diabetic cardiomyopathy model was established using a multiple low‐dose STZ protocol [54]. Briefly, male C57BL/6 mice were 7 weeks old at the start of modeling, so that subsequent interventions could begin at 8 weeks of age. After a 12 h fast, mice received intraperitoneal injections of STZ (50 mg/kg/day; Sigma‐Aldrich) freshly dissolved in 0.1 M citrate buffer (pH 4.5) for 5 consecutive days. Control (CON) mice were injected intraperitoneally with an equal volume of citrate buffer on the same schedule. Blood glucose was measured using a handheld glucometer 3 days after the final STZ injection, and mice with blood glucose > 11.1 mmol/L were considered diabetic and included in subsequent experiments. No high‐fat diet was used in this study.

After 8 weeks of diabetes progression, cardiac function was evaluated by transthoracic echocardiography, and DCM was confirmed primarily by impaired systolic function parameters, including left ventricular ejection fraction (EF) and fractional shortening (FS), together with structural indices such as left ventricular internal diameter at end‐systole (LVIDs) and left ventricular internal diameter at end‐diastole (LVIDd).

### 5.5 Intermittent Fasting Intervention

8‐week‐old male C57BL/6 mice (n = 24) were randomly assigned in equal numbers to three groups: CON, DCM, and DCM‐IF. Control (CON) mice received intraperitoneal injections of an equal volume of citrate buffer (0.1 M, pH 4.5) for 5 consecutive days, following the same injection schedule as the STZ protocol, and were maintained under ad libitum feeding. STZ‐induced DCM mice were maintained under ad libitum feeding (DCM) or subjected to IF (DCM‐IF). Mice in the IF group underwent 24 h fasting every other day for 8 weeks, while DCM mice were maintained with ad libitum access to food and water. Body weight, food intake, and water intake were recorded throughout the intervention period.

### 5.6 Antibiotics Treatment and Fecal Microbiota Transplantation

To investigate the contribution of the gut microbiota to IF‐mediated cardioprotection, 8‐week‐old male C57BL/6 mice (n = 30) were randomly assigned to five groups: CON, DCM, DCM‐IF, DCM‐IF+antibiotics (DCM‐IF+ABX), and DCM+FMT.

Recipient mice for FMT (DCM+FMT) were pretreated with an antibiotic cocktail for 1 week to deplete endogenous gut microbiota and establish pseudo–germ‐free conditions. Vancomycin (50 mg/kg), neomycin (100 mg/kg), metronidazole (100 mg/kg), and amphotericin B (1 mg/kg) were freshly prepared in sterile water and administered by oral gavage once daily, whereas ampicillin sodium was supplied in drinking water at 1 g/L throughout the pretreatment period, following published protocols [55, 56]. After antibiotic pretreatment, mice were allowed a 2‐day washout period prior to FMT. For FMT donors, fecal pellets were collected from DCM‐IF mice at the end of the IF intervention. Fresh fecal pellets were suspended in sterile saline at 100 mg feces per 1 mL, vortexed/mixed for 10 min, and centrifuged at 800 g for 3 min to remove debris. The supernatant was further centrifuged at 15 000 g for 5 min, the supernatant discarded, and the pellet resuspended in 1 mL sterile saline. The final suspension was prepared and administered to recipients within 10 min of preparation. Recipient mice were gavaged with 200 µL of the fecal bacterial suspension every 2 days for a total duration of 7 weeks [57].

To maintain microbiota depletion during IF, mice in the DCM‐IF+ABX group received a broad‐spectrum antibiotic cocktail in drinking water. Antibiotic water was initiated 1 week before the IF intervention and maintained throughout the 8‐week IF period. Because the short‐term high‐intensity pretreatment regimen used for establishing pseudo–germ‐free recipients is not optimal for prolonged administration and may compromise animal health and confound metabolic readouts, we adopted a long‐term depletion protocol reported by Liu et al. [29]. Briefly, antibiotics were supplied ad libitum in sterile drinking water containing penicillin G sodium (0.4 g/L), metronidazole (0.4 g/L), neomycin sulfate (0.4 g/L), streptomycin sulfate (0.4 g/L), and vancomycin hydrochloride (0.25 g/L) throughout the treatment period.

### 5.7 A. *Muciniphila* Supplementation

*A. muciniphila* (BeNa Culture Collection, BNCC341917) was cultured anaerobically at 37°C in fluid thioglycolate medium (BeNa Culture Collection, BNCC353538) and authenticated by full‐length 16S rDNA sequencing. For gavage, bacteria were freshly harvested by centrifugation at 15,000 g for 5 min, and the pellet was resuspended in sterile PBS to a final concentration of 5 × 10^9^ CFU/mL. This dosing strategy was selected based on previous in vivo studies reporting oral supplementation of *A. muciniphila* at 1 × 10^9^ CFU per animal per day in 200 µL [45, 58]. 8‐week‐old male C57BL/6 mice (n = 18) were randomly assigned to three groups: CON, DCM, and DCM+AKK. Mice in the DCM+AKK group received 200 µL of *A. muciniphila* suspension (1 × 10^9^ CFU per mouse) by oral gavage once daily for 8 weeks. In parallel, mice in the CON and DCM groups were gavaged with an equal volume of sterile PBS on the same schedule.

### 5.8 1‐Methyl‐L‐Histidine Administration

To evaluate the cardioprotective effects of the microbiota‐associated metabolite 1‐methyl‐L‐histidine, 8‐week‐old male mice (n = 18) were randomly assigned to three groups: CON, DCM, and DCM+1‐methyl‐L‐histidine (DCM+His). 1‐methyl‐L‐histidine was dissolved in sterile saline, and mice in the DCM+His group received 1‐methyl‐L‐histidine by oral gavage at 50 mg/kg every 2 days for 8 weeks. In parallel, mice in the CON and DCM groups were gavaged with an equal volume of sterile saline on the same schedule.

### 5.9 Echocardiography

Mice were anesthetized with 2%–3% isoflurane for induction and maintained at 0.5%–1% isoflurane during imaging. Transthoracic echocardiography was performed using a Vevo 3100 high‐resolution ultrasound system (Visual Sonics, Canada) equipped with a 15 MHz linear‐array transducer. M‐mode images were acquired in the parasternal short‐axis view at the level of the papillary muscles. EF%, FS%, LVIDs, and LVIDd were calculated using Vevo LAB software. Body temperature and heart rate (maintained at ∼450 bpm) were continuously monitored throughout imaging.

### 5.10 Histological Analysis

Hearts were harvested, rinsed in cold PBS, fixed in 4% paraformaldehyde, paraffin‐embedded, and sectioned at 5 µm. Sections were deparaffinized in xylene and rehydrated through graded ethanol. Cardiomyocyte cross‐sectional area (CSA) was evaluated by wheat germ agglutinin (WGA) staining (Beyotime, AL‐1023‐2). Briefly, sections were incubated with WGA working solution (1:100) for 1 h at 37°C in the dark, washed with PBS, and counterstained with DAPI (Sigma‐Aldrich, 9542). CSA was quantified using ImageJ. For each heart, 50 cardiomyocytes were measured per section, and values were averaged to obtain one biological replicate per animal (n = 3 mice per group). Myocardial fibrosis was assessed by Sirius red staining (Ybio, YB10278). Dewaxed and rehydrated sections were incubated with Sirius red solution for 40 min at room temperature, followed by washing according to the manufacturer's instructions. Fibrosis area was quantified using ImageJ as the percentage of Sirius red–positive area. For each heart, three randomly selected fields were analyzed and averaged.

For immunohistochemical staining, sections were incubated overnight at 4°C with primary antibodies against 4‐hydroxynonenal (4‐HNE; Abcam, ab48506; 1:200) or 8‐hydroxy‐2′‐deoxyguanosine (8‐OHdG; Santa Cruz Biotechnology, sc‐393871; 1:100). On the next day, sections were incubated with the appropriate secondary antibody at 37°C for 1 h, washed three times with PBS (5 min each), and developed with 3,3′‐diaminobenzidine (DAB). Images were acquired using an Olympus BX53 microscope.

### 5.11 Western Blot Analysis

Total protein was extracted from 20 mg of frozen left ventricular tissue using RIPA lysis buffer, and protein concentration was determined with a BCA protein assay kit. Equal amounts of protein (50 µg per lane) were separated by SDS‐PAGE and transferred onto PVDF membranes. Membranes were blocked with 5% non‐fat milk and incubated overnight at 4°C with primary antibodies against α‐smooth muscle actin (α‐SMA, ProteinTech, 67735‐1‐Ig), caspase‐3 (Abcam, ab184787), cleaved caspase‐3 (Cell Signaling Technology, #9664), BCL2 (Abcam, ab32124), BAX (ProteinTech, 50599‐2‐1 g), and GAPDH (ProteinTech, 10494‐1‐AP). After incubation with appropriate HRP‐conjugated secondary antibodies, immunoreactive bands were detected using an enhanced chemiluminescence substrate and imaged on a ChemiDoc MP imaging system.

### 5.12 Real‐Time Polymerase Chain Reaction (RT‐PCR) Analysis

Total RNA was isolated from 10 mg of frozen left ventricular tissue using RNAiso Plus (Takara, 9108, Japan) according to the manufacturer's instructions. For reverse transcription, 1 µg of total RNA was used to synthesize cDNA with HiScript III RT SuperMix (Vazyme, R323‐01, China). Quantitative PCR was performed on a LightCycler 480 system (Roche, Switzerland) using gene‐specific primers, with 2 µL of diluted cDNA template added to each reaction. Relative mRNA expression levels were calculated using the 2^−ΔΔCt^ method, with *Gapdh* used as the internal reference gene. Primer sequences are provided in Table S2.

### 5.13 Malondialdehyde Measurement

Cardiac lipid peroxidation was quantified by measuring malondialdehyde (MDA) in 20 mg of frozen left ventricular tissue using a commercial Lipid Peroxidation MDA Assay Kit (Beyotime, S0131S) according to the manufacturer's instructions. Briefly, LV tissue was homogenized in the lysis buffer provided with the kit on ice and centrifuged to obtain clarified supernatants. MDA levels were determined based on the thiobarbituric acid reaction, and the absorbance of the resulting chromogenic adduct was measured using a microplate reader. MDA concentrations were calculated from a standard curve, normalized to total protein content determined by a BCA assay, and expressed as nmol/mg protein.

### 5.14 Metagenomic Sequencing Analysis

Fecal pellets were collected at the end of the 8‐week intervention period and prior to echocardiographic assessment, placed into sterile tubes, immediately snap‐frozen, and stored at −80°C until processing. Microbial genomic DNA was extracted using a stool DNA isolation kit (Tiangen Biotech, Beijing, China; DP328) according to the manufacturer's instructions. DNA quantity and integrity were assessed prior to library construction. For each sample, ∼1 µg DNA was fragmented to an average insert size of ∼350 bp by sonication, and sequencing libraries were prepared using the NEBNext Ultra DNA Library Prep Kit for Illumina (NEB, USA) with unique index barcodes. End repair, A‐tailing, adaptor ligation, and PCR enrichment were performed following the kit protocol. Libraries were purified using AMPure XP beads (Beckman Coulter) and sequenced on an Illumina HiSeq 2500 platform (paired‐end 150 bp) at Novogene (Beijing, China).

Raw reads were quality‐filtered to remove adaptor contamination and low‐quality sequences using Readfq (v8.0), generating clean reads for downstream analysis. Clean reads were de novo assembled into scaffolds using SOAPdenovo (v2.04). Open reading frames (ORFs) were predicted with MetaGeneMark (v2.10), and a non‐redundant gene catalog was constructed by removing redundant sequences using CD‐HIT (v4.5.8). Taxonomic annotation of unigenes was performed by DIAMOND (v0.9.9.110) against the NCBI NR database, and assignments were summarized using a lowest common ancestor (LCA) approach. Functional annotation was obtained by mapping unigenes to KEGG Orthology (KO) entries based on DIAMOND alignments, and KO profiles were subsequently summarized into KEGG pathways. Alpha and beta diversity were calculated from taxonomic abundance tables. Beta diversity was assessed using Bray‐Curtis distances and visualized by principal coordinates analysis (PCoA). Group differences in beta diversity were assessed by PERMANOVA. Differential taxonomic abundance analysis was performed between groups using taxonomic abundance profiles. Raw *p* values were adjusted for multiple comparisons using the Benjamini–Hochberg false discovery rate (FDR) method. Taxa were considered differentially abundant when FDR < 0.05 and |Log2(fold change)| > 2. LEfSe analysis was performed to identify discriminative taxa among groups, with an LDA score > 4 used as the cutoff.

### 5.15 Untargeted Metabolomics

Untargeted metabolomic analyses of serum and left ventricular tissue were performed as previously described with minor modifications [59, 60]. For serum extraction, 50 µL serum was mixed with 300 µL methanol, vortexed thoroughly, and centrifuged at 12 000 rpm for 15 min at 4°C. The supernatant was collected and dried under a nitrogen stream. For tissue extraction, 20 mg of frozen LV tissue was homogenized in 400 µL methanol, followed by centrifugation at 12 000 rpm for 15 min at 4°C. The supernatant was similarly collected and dried under nitrogen. Dried extracts from both serum and tissue samples were reconstituted in 100 µL acetonitrile/water (1:1, v/v) for ultra‐performance liquid chromatography–mass spectrometry (UPLC–MS) analysis.

Metabolomic profiling in both positive and negative electrospray ionization modes (ESI^+^ and ESI^−^) was performed using a SHIMADZU CBM‐30A Lite LC system (Shimadzu Corporation, Kyoto, Japan) coupled to an API 6600 Q‐Triple TOF mass spectrometer (AB SCIEX, Foster City, CA, USA). A 2 µL aliquot of each sample was injected onto an XBridge BEH Glycan amide column (4.6 × 100 mm, 3.5 µm). The mobile phases consisted of 5 mM ammonium acetate and 0.1% formic acid in water (A) and acetonitrile (B). The gradient started at 98% B for 0.5 min, decreased linearly to 40% B at 13 min, and then returned to 98% B by 16 min for re‐equilibration. The column temperature was maintained at 40°C, and the collision energy was set at 40 V. Quality control (QC) samples were prepared by pooling 10 µL aliquots from each sample and were injected once every 8 runs throughout the analytical sequence to monitor instrument stability and data reproducibility.

Data preprocessing was performed using the XCMS software as described previously [59, 60]. Principal component analysis (PCA) was performed to show the metabolic diversity between the CON, DCM, and DCM‐IF groups. Univariate statistical data analysis was performed through the online website (https://www.omicshare.com/tools/) to assess the significance of each feature. For each pairwise comparison, raw *p*‐values were adjusted for multiple testing across all detected metabolic features using the Benjamini–Hochberg false discovery rate (FDR) method. Metabolites with FDR < 0.05 were considered differential metabolites and were used for downstream overlap analysis. Differential metabolites shared between the CON vs. DCM and DCM vs. DCM‐IF comparisons in serum and left ventricular tissue were further intersected to identify IF‐responsive candidate metabolites. These candidates were then subjected to metabolite identification based on accurate mass, product ion spectra, relevant literature, and online databases, including the Human Metabolome Database and metabolite identification and dysregulated network analysis resources.

### 5.16 Targeted Metabolomics and Lipidomics

Targeted metabolomic and lipidomic analyses were performed using serum, feces, and left ventricular tissue samples from mice. Samples were extracted with H2O/MeOH (1:1, v/v). Targeted analyses were carried out on a SHIMADZU CBM‐30A Lite LC system coupled to an API 6500 QTRAP mass spectrometer (AB SCIEX) operated in multiple reaction monitoring (MRM) mode. Chromatographic separation was performed on a Waters Acquity HSS T3 column (2.1 × 100 mm, 1.8 µm) using 0.1% formic acid in water as mobile phase A and acetonitrile as mobile phase B. All targeted metabolite transitions were optimized using authentic standards, and analyses were conducted in positive electrospray ionization mode. The gradient program was as follows: 0–0.5 min, 2% B; 0.5–4 min, 2%–40% B; 4–8 min, 40%–95% B; 8–8.5 min, 95% B; and 8.5–12 min, 95%–2% B. Other instrumental conditions were identical to those used for the untargeted metabolomics analysis.

A targeted method was developed for quantitative analysis of *N*‐2‐fluorenylacetamide (Aladdin, F106909, China), ergothioneine (Aladdin, L134175, China), orotate (Aladdin, O137322, China), tiglyl carnitine (Macklin, T878002, China), valerylcarnitine (Aladdin, V648609, China), 1‐methyl‐L‐histidine (Aladdin, M112851, China), indole‐3‐lactic Acid (Aladdin, I157602, China), pantothenic acid (Aladdin, P275366, China), and L‐histidine (Aladdin, H108261, China). Furthermore, lipid levels were evaluated by MRM using 1,2‐dipalmitoyl‐sn‐glycero‐3‐phosphocholine (PC 16:0) (Aladdin, D130424, China), 1,2‐dioleoyl‐sn‐glycero‐3‐phosphoethanolamine (PE 18:1) (Aladdin, D155634, China), and Fatty acids (FA 18:1 and FA 16:0) (Aladdin, O108485 and P101061, China) as internal standard (200 ng/mL). The methods were linear over a range of 1–1000 ng/mL.

### 5.17 In Vitro Mixed‐Microbial Transformation

Mixed microbial communities were prepared from freshly collected mouse and cultured anaerobically in modified Gifu Anaerobic medium at 37°C [61]. For precursor‐conversion assays, mixed microbial cultures were supplemented with 100 µM L‐histidine (Aladdin, H108261, China) or 100 µM L‐anserine (Aladdin, L292670, China) and incubated for 24 h at 37°C under anaerobic conditions. Microbial growth was monitored by measuring OD600, and metabolite levels were normalized to OD600 where indicated. After incubation, cultures were centrifuged to remove microbial cells, and the resulting supernatants were collected and mixed with 200 µL of 50% methanol for LC–MS detection of 1‐methyl‐L‐histidine. The composition of the modified Gifu Anaerobic medium is provided in Table S1 [61].

### 5.18 In Vitro Single‐Microbial Transformation

*A. muciniphila* (BeNa Culture Collection, BNCC341917) was cultured anaerobically in fluid thioglycolate medium (BeNa Culture Collection, BNCC353538) at 37°C. For precursor‐conversion assays, freshly prepared *A. muciniphila* cultures were supplemented with 100 µM L‐histidine or 100 µM L‐anserine and incubated anaerobically for 24 h at 37°C. Bacterial growth was monitored by measuring OD600, and 1‐methyl‐L‐histidine levels were normalized to OD600 where indicated. After incubation, cultures were centrifuged to remove bacterial cells, and the supernatants were collected for LC‐MS detection of 1‐methyl‐L‐histidine.

### 5.19 Statistical Analysis

Statistical analyses were performed using GraphPad Prism and R software. Data are presented as mean ± SD unless otherwise indicated. For comparisons between two groups, an unpaired two‐tailed Student's *t*‐test was used, whereas the Mann–Whitney U test was applied when nonparametric analysis was more appropriate. For comparisons among three or more groups, one‐way ANOVA followed by Dunnett's multiple‐comparisons test was used, whereas the Kruskal‐Wallis test with Dunn's post hoc test was applied for nonparametric data where appropriate. Correlations were assessed using Pearson or Spearman analysis as indicated in the corresponding figure legends. Beta‐diversity of the gut microbiota was evaluated by PCoA based on Bray‐Curtis distances, and group differences were assessed by PERMANOVA. Where indicated, supplementary PERMANOVA analyses were additionally performed with *Akkermansia* abundance included as a covariate to evaluate its contribution to community variation. All quantified data represent biological replicates unless otherwise specified. Investigators were blinded to group allocation during echocardiographic and histological quantification. A two‐sided *p* < 0.05 was considered statistically significant.

## Author Contributions

H.C.G. made substantial contributions to the conception and design of the study. K.Y.J., F.X., Y.W.P., L.F.M., X.R.W., Y.R.X., and T.T. contributed to the animal experiments and acquired experimental research data. K.Y.J. and F.X. participated in the analysis of research data. K.Y.J., H.C.G., and F.X. drafted the manuscript and critically revised it for important intellectual content. All authors have read and agreed to the submitted version of the manuscript.

## Conflicts of Interest

The authors declare no conflicts of interest.

## Supporting information




**Supporting File**: advs76528‐sup‐0001‐SuppMat1.docx.

## Data Availability

The raw metagenomic sequencing data generated in this study have been deposited in the NCBI BioProject database under accession number PRJNA1464059. All other data supporting the findings of this study are available within the article and its Supplementary Information, or from the corresponding authors upon reasonable request.
