# Supplementary material for: Intermittent Fasting Restores Cardiac Lipid Homeostasis in Diabetic Cardiomyopathy in Association With Akkermansia Muciniphila and 1‐methyl‐L‐histidine
Source: Adv Sci (Weinh). 2026 Jul 10;13(55):e76528. doi: 10.1002/advs.76528 (PMC13353179; doi:10.1002/advs.76528)
Supplement: Supplementary file 1 [file ADVS-13-e76528-s001.docx]

**Supplementary information**

**Intermittent fasting restores cardiac lipid homeostasis in diabetic cardiomyopathy in association with *Akkermansia muciniphila* and 1-methyl-L-histidine**

Kaiyuan Jiang^#1,2^, Fen Xiong^#1,2^, Yiwen Peng^1^, Lingfei Meng^1^, Xinran Wang^1^, Yingruo Xu^1^, Tian Tang^1^, Hongchang Gao*^1,2^

^1^Institute of Metabonomics & Medical NMR, School of Pharmaceutical Sciences, Wenzhou Medical University, Wenzhou 325035, China.

^2^Oujiang Laboratory (Zhejiang Lab for Regenerative Medicine, Vision and Brain Health); Scientific Research Center of Wenzhou Medical University, Wenzhou 325035, China.

*Corresponding authors.

E-mail: gaohc27@wmu.edu.cn (Hongchang Gao)

**Table S1. Composition of the GB medium used for in vitro microbial culture**

| Composition Name | Concentration (g/L) |
| --- | --- |
| Dextrose | 0.33 |
| Soluble starch | 3.25 |
| Yeast extract | 3.38 |
| Meat extract | 4.06 |
| Liver extract | 0.78 |
| Digested serum | 6.50 |
| Peptone | 3.25 |
| Soya peptone | 1.95 |
| Proteose peptone | 3.25 |
| L-Tryptophan | 0.13 |
| L-Arginine | 0.65 |
| L-Cysteine Hydrochloride | 0.37 |
| Casein enzymic hydrolysate | 5.25 |
| Potassium dihydrogen phosphate | 1.63 |
| Sodium thioglycolate | 0.20 |
| Sodium chloride | 1.95 |
| Sodium acetate | 1.75 |
| Vitamin K1 | 0.00325 |
| Hemin | 0.0065 |
| Resazurin | 0.0009 |

**Table S2. List of primers used for qPCR.**

| Gene | Forward primer (5’ – 3’) | Reverse primer (5’ – 3’) |
| --- | --- | --- |
| *A.muciniphila* | GTGAGGTAACGGCTCACCAA | GGACCGTGTCTCAGTTCCAG |
| 16S | TCCTACGGGAGGCAGCAGT | GACTACCAGGGTATCTAATCCTGTT |
| *Mouse-ANP* | GGGTAGGATTGACAGGATTGG | CTCCTTGGCTGTTATCTTCGG |
| *Mouse-BNP* | TGGGAGGTCACTCCTATCCT | GGCCATTTCCTCCGACTTT |
| *Mouse-Acly* | AAGGAGCCATTGTACCTGCC | ATCAAACCCAGCTCTCTGGC |
| *Mouse-Acss2* | GTCCCCACCAGTTAAGAGGC | TGCCACCATAAGTCAACCCC |
| *Mouse-Acaca* | ATTGGGGCTTACCTTGTCCG | GCACCTGCTCCTGTCAGAAT |
| *Mouse-Acacb* | CACCCAACTCTGAAGGGGAC | CCAGGGTGCCATGATTTCCT |
| *Mouse-Fasn* | CAAGTGTCCACCAACAAGCG | GGAGCGCAGGATAGACTCAC |
| *Mouse-Scd1* | CCGGAGACCCCTTAGATCGA | TAGCCTGTAAAAGATTTCTGCAAACC |
| *Mouse-Fads1* | TGGCACCTCGACATGGAATC | TTGATATCGGGGTCCTTGCG |
| *Mouse-Fads2* | CCTCCTGTCCCACATCATCG | GAGGACAAAGGCTGTGACGA |
| *Mouse-Fads3* | CTGATCGAGGACTTCCGAGC | AGGCCAACAACTCCATAGCC |
| *Mouse-Elovl1* | GTGGCCCAGCCCTACCTT | TGTGCAGTGAGACCAGGACAA |
| *Mouse-Elovl2* | TCAATGCTTTCTTGGACAACATG | GGTAAGAGTCCAGCAGGAACCA |
| *Mouse-Elovl3* | TTCTCACGCGGGTTAAAAATG | GGGCCTTAAGTCCTGAAACGT |
| *Mouse-Elovl5* | ATGGACACCTTTTTCTTCATCCTT | ATGGTAGCGTGGTGGTAGACATG |
| *Mouse-Elovl6* | CAGCAAAGCACCCGAACTA | AGGAGCACAGTGATGTGGTG |
| *Mouse-Gpat1* | CATCCTCGTCATACCCGTGG | GCTCTCGTTCTTCTTGGGCT |
| *Mouse-Gpat2* | AAGAAAGAGGTACAGCGTATCC | GTGGAGAGCCCTCCTGCACAG |
| *Mouse-Gpat3* | CTTTGAAATCGGAGGAACCA | TTTGCAAACTGAACTGCGTC |
| *Mouse-Gpat4* | GGCATGGTGACGTACCTTCT | GCTTCAGCCAGCACAAGAC |
| *Mouse-Agpat1* | GCTGGCTGGCAGGAATCAT | GTCTGAGCCACCTCGGACAT |
| *Mouse-Agpat2* | TTTGAGGTCAGCGGACAGAA | AGGATGCTCTGGTGATTAGAGATGA |
| *Mouse-Agpat3* | GGAGGAAAACACCTGTCCAC | TCAAGGGTGTCCGACCTG |
| *Mouse-Agpat5* | CTAGCGAATCATCAAAGCACA | TCTTTCAGTACGTAGCGCACA |
| *Mouse-Lpin1* | CCTTCTATGCTGCTTTTGGGAACC | GTGATCGACCACTTCGCAGAGC |
| *Mouse-Lpin2* | AGTTGACCCCATCACCGTAG | CCCAAAGCATCAGACTTGGT |
| *Mouse-Lpin3* | TGGAATTGGGATGACAAGGT | CACTGCAAGTACCCCTTGGT |
| *Mouse-Dgat1* | TCGTGGTATCCTGAATTGGTG | AGGTTCTCTAAAAATAACCTTGCATT |
| *Mouse-Dgat2* | GGCGCTACTTCCGAGACTAC | TGGTCAGCAGGTTGTGTGTC |
| *Mouse-Gapdh* | GCACAGTCAAGGCCGAGAAT | GCCTTCTCCATGGTGGTGAA |

**Table S3. Genus-level PERMANOVA of Bray–Curtis β-diversity with *Akkermansia* abundance included as a covariate.**

PERMANOVA was performed using genus-level relative abundance profiles based on Bray–Curtis distances. The overall group effect was first assessed across CON, DCM, and DCM-IF mice, followed by models incorporating *Akkermansia* abundance as a covariate. Marginal effects were calculated to evaluate the independent contributions of group and *Akkermansia* abundance to community variation. R² values indicate the proportion of variance explained.

| **Model** | **Term** | **Df** | **R^2^** | **F** | **P** |
| --- | --- | --- | --- | --- | --- |
| Model 1 | Group | 2 | 0.339 | 3.853 | 0.004 |
| Model 2 | Overall model: *Akkermansia* abundance + Group | 3 | 0.433 | 3.568 | 0.003 |
| Model 3 | Overall model: Group + *Akkermansia* abundance | 3 | 0.433 | 3.568 | 0.001 |
| Model 4 | Group (marginal effect) | 2 | 0.216 | 2.672 | 0.031 |
| Model 4 | *Akkermansia* abundance (marginal effect) | 1 | 0.094 | 2.320 | 0.080 |

Notes:

Model 1, PERMANOVA with group as the only explanatory variable.

Model 2, sequential PERMANOVA with *Akkermansia* abundance entered before group.

Model 3, sequential PERMANOVA with group entered before *Akkermansia* abundance.

Model 4, marginal PERMANOVA testing the independent effects of group and *Akkermansia* abundance.

**Table S4.** **FDR-filtered differential metabolites in serum among CON, DCM, and DCM-IF groups.**

| **m/z** | **Ret. Time** | **Name** | **Molecular formula** | **adduct** | **Log2(FC)**  **CON vs DCM** | **P value**  **CON vs DCM** | **FDR**  **CON vs DCM** | **Log2(FC)**  **DCM vs DCM-IF** | **P value**  **DCM vs DCM-IF** | **FDR**  **DCM vs DCM-IF** |
| --- | --- | --- | --- | --- | --- | --- | --- | --- | --- | --- |
| 224.1094 | 8.61 | N-2-fluorenylacetamide | C_7_H_14_N_2_O_3_S | M+H | 0.48 | 0.01248 | 0.04973 | -0.66 | 0.00247 | 0.03324 |
| 112.0861 | 11.68 | Histamine | C_5_H_9_N_3_ | M+H | 0.62 | 0.001226 | 0.01446 | -0.38 | 0.01086 | 0.04969 |
| 157.0811 | 9.75 | L-Histidine | C_6_H_9_N_3_O_2_ | M+H | 0.43 | 0.00986 | 0.04466 | -0.71 | 0.00246 | 0.03324 |
| 198.0948 | 8.78 | L-Citrulline | C_6_H_13_N_3_O_3_ | M+Na | 1.27 | 1.022E-08 | 2.657E-06 | -0.44 | 0.01290 | 0.04936 |
| 230.0939 | 8.97 | Ergothioneine | C_9_H_15_N_3_O_2_S | M+H | 0.68 | 0.00784 | 0.04817 | -1.03 | 0.00058 | 0.01701 |
| 170.0930 | 11.25 | 1-Methyl-L-histidine | C_7_H_11_N_3_O_2_ | M+H | -0.37 | 0.00994 | 0.03017 | 1.00 | 0.00259 | 0.04920 |
| 102.0538 | 8.91 | N,N-Dimethylglycine | C_4_H_9_NO_2_ | M-H | 0.46 | 0.01172 | 0.04976 | -0.76 | 0.00305 | 0.03698 |
| 168.0299 | 6.68 | N-(2-Furoyl)glycine | C_7_H_7_NO_4_ | M-H | 0.48 | 0.01248 | 0.04973 | -0.66 | 0.00247 | 0.03324 |
| 243.1012 | 5.23 | Prolylphenylalanine | C_14_H_18_N_2_O_3_ | M-H_2_0-H | 0.74 | 0.00032 | 0.00648 | -0.60 | 0.00334 | 0.03813 |
| 228.1899 | 3.46 | Valerylcarnitine | C_12_H_23_NO_4_ | M+H-H_2_O | -0.21 | 0.00479 | 0.04953 | 0.30 | 0.00389 | 0.04920 |
| 226.1831 | 3.26 | Tiglylcarnitine | C_12_H_21_NO_4_ | M+H-H_2_O | -0.27 | 0.02878 | 0.04096 | 0.36 | 0.00668 | 0.04810 |
| 201.1015 | 5.46 | N-Acetylserotonin | C_12_H_14_N_2_O_2_ | M-H_2_O+H | 0.46 | 0.01172 | 0.044976 | -0.76 | 0.00305 | 0.03698 |
| 206.1986 | 8.94 | Indolelactic acid | C_11_H_11_NO_3_ | M+H | 0.63 | 0.00881 | 0.04999 | -0.99 | 0.00057 | 0.01701 |
| 213.0150 | 8.96 | 5-Deoxyribose-1-phosphate | C_5_H_11_O_7_P | M-H | 0.97 | 1.02E-08 | 1.29E-06 | -0.46 | 0.00054 | 0.02289 |
| 155.0176 | 6.54 | Orotate | C_5_H_4_N_2_O_4_ | M-H | -0.21 | 0.02513 | 0.04966 | 0.69 | 0.00190 | 0.04363 |
| 220.1182 | 8.46 | Pantothenic acid | C_9_H_17_NO_5_ | M+H | 0.44 | 0.01585 | 0.04955 | -0.68 | 0.00501 | 0.04845 |

**Notes:** Raw *P* values were adjusted using the Benjamini–Hochberg false discovery rate (FDR) method within each pairwise comparison. The table lists metabolites that met the FDR < 0.05 criterion in the corresponding comparisons and were used for downstream overlap analysis.

**Table S5. FDR-filtered differential metabolites in heart tissue among CON, DCM, and DCM-IF groups.**

| **m/z** | **Ret. Time** | **Name** | **Molecular formula** | **adduct** | **Log2(FC)**  **CON vs DCM** | **P value**  **CON vs DCM** | **FDR**  **CON vs DCM** | **Log2(FC)**  **DCM vs DCM-IF** | **P value**  **DCM vs DCM-IF** | **FDR**  **DCM vs DCM-IF** |
| --- | --- | --- | --- | --- | --- | --- | --- | --- | --- | --- |
| 124.0873 | 11.39 | L-Histidinol | C_6_H_11_N_3_O | M-H_2_O+H | -0.23 | 0.01136 | 0.03423 | 0.37 | 0.00249 | 0.02870 |
| 170.0919 | 11.39 | 1-Methyl-L-histidine | C_7_H_11_N_3_O_2_ | M+H | -0.27 | 0.02417 | 0.04040 | 0.51 | 0.00321 | 0.03200 |
| 147.067 | 9.29 | L-Glutamine | C_5_H_10_N_2_O_3_ | M-H | -0.34 | 0.00587 | 0.01989 | 0.34 | 0.00630 | 0.04860 |
| 157.0826 | 9.86 | L-Histidine | C_6_H_9_N_3_O_2_ | M+H | -0.44 | 9.04E-05 | 0.00113 | 0.26 | 0.01546 | 0.04868 |
| 164.0791 | 8.37 | L-Phenylalanine | C_9_H_11_NO_2_ | M-H | -1.01 | 0.00534 | 0.01967 | 1.47 | 1.98E-05 | 0.00229 |
| 228.0788 | 9.21 | Ergothioneine | C_9_H_15_N_3_O_2_S | M-H | 1.53 | 3.93E-06 | 7.79E-05 | -1.76 | 2.48E-06 | 0.00064 |
| 171.0974 | 11.42 | N(pi)-Methyl-L-histidine | C_7_H_11_N_3_O_2_ | M+H | -0.26 | 0.00950 | 0.02988 | 0.43 | 0.00197 | 0.02541 |
| 198.1841 | 3.26 | N-Nonanoylglycine | C_11_H_21_NO_3_ | M+H-H_2_O | -1.11 | 0.01352 | 0.03915 | 1.44 | 0.00036 | 0.01133 |
| 168.0318 | 7.00 | Hypotaurine | C_2_H_7_NO_2_S | M+CH_3_COO | 0.84 | 1.98E-08 | 2.16E-06 | -0.36 | 0.00043 | 0.01099 |
| 175.1466 | 3.26 | Ethyl lysine | C_8_H_18_N_2_O_2_ | M+H | -0.48 | 0.00010 | 0.00120 | 0.38 | 0.00044 | 0.01264 |
| 144.1016 | 8.62 | Proline betaine | C_7_H_13_NO_2_ | M+H | 0.80 | 0.00720 | 0.02464 | -0.93 | 0.00396 | 0.03454 |
| 91.0551 | 5.24 | Iminoglycine | C_2_H_3_NO_2_ | M+NH_4_ | -0.23 | 1.77E-05 | 0.00035 | 0.17 | 0.00131 | 0.02113 |
| 123.0936 | 2.96 | Phenethylamine | C_8_H_11_N | M+H | -0.25 | 0.00032 | 0.00269 | 0.20 | 0.00022 | 0.00908 |
| 169.0167 | 6.91 | Gallic acid | C_7_H_6_O_5_ | M-H | -4.02 | 2.45E-08 | 2.16E-06 | 2.11 | 0.00097 | 0.01757 |
| 311.2202 | 4.28 | (8E,10S,12Z,15Z)-10-Hydroperoxyoctadeca-8,12,15-trienoate | C_18_H_30_O_4_ | M+H | -1.21 | 1.57E-05 | 0.00031 | 0.92 | 0.00039 | 0.01186 |
| 321.2399 | 3.39 | (19S)-Hydroxyeicosatetraenoic acid | C_20_H_32_O_3_ | M+H | -0.82 | 0.00188 | 0.00894 | 0.99 | 0.00148 | 0.02218 |
| 97.027 | 9.80 | Propionic acid | C_3_H_6_O_2_ | M+H | -0.29 | 0.00162 | 0.00809 | 0.19 | 0.00091 | 0.01809 |
| 225.1965 | 14.62 | Tetradec-5-ynoic acid | C_14_H_24_O_2_ | M+NH_4_ | -0.13 | 0.00532 | 0.01963 | 0.12 | 0.00018 | 0.00804 |
| 226.1799 | 3.26 | Tiglylcarnitine | C_12_H_21_NO_4_ | M+H-H_2_O | -1.45 | 0.00990 | 0.03078 | 1.83 | 0.00070 | 0.01527 |
| 228.1953 | 3.46 | Valerylcarnitine | C_12_H_23_NO_4_ | M+H-H_2_O | -1.00 | 0.00837 | 0.02732 | 1.30 | 0.00042 | 0.01236 |
| 303.2314 | 5.96 | Arachidonic acid | C_20_H_32_O_2_ | M-H | 0.55 | 0.00100 | 0.00504 | -0.48 | 0.00526 | 0.04428 |
| 204.106 | 11.62 | L-Acetylcarnitine | C_9_H_17_NO_4_ | M+H | 0.71 | 4.58E-08 | 2.83E-06 | -0.48 | 7.12E-05 | 0.00361 |
| 211.1678 | 3.26 | Methyl (2E,6Z)-dodecadienoate | C_9_H_17_NO_4_ | M+H | -1.14 | 0.00941 | 0.02968 | 1.49 | 0.00051 | 0.01286 |
| 219.0753 | 8.45 | 5-Hydroxy-L-tryptophan | C_11_H_12_N_2_O | M-H | -0.82 | 0.00020 | 0.00159 | 0.93 | 0.00048 | 0.01162 |
| 206.1969 | 9.16 | Indolelactic acid | C_11_H_11_NO_3_ | M+H | 1.08 | 6.62E-07 | 3.16E-05 | -1.27 | 1.44E-05 | 0.00220 |
| 111.0063 | 10.10 | 2,5-Dioxopentanoate | C_5_H_6_O_4_ | M-H_2_O-H | 0.46 | 8.06E-09 | 2.06E-06 | -0.27 | 3.30E-05 | 0.00249 |
| 224.1094 | 8.61 | N-2-fluorenylacetamide | C_7_H_14_N_2_O_3_S | M+H | 1.53 | 3.66E-06 | 0.00010 | -0.59 | 0.00589 | 0.04344 |
| 60.0438 | 5.21 | 2-Aminoacetaldehyde | C_2_H_5_NO | M+H | -0.13 | 0.01671 | 0.04507 | 0.14 | 0.00342 | 0.03281 |
| 347.0375 | 6.82 | Inosine-5-phosphate | C_10_H_13_N_4_O_8_P | M-H | 0.31 | 0.01134 | 0.03308 | -0.36 | 0.00280 | 0.03094 |
| 226.0897 | 5.86 | 2-Deoxycytidine | C_9_H_13_N_3_O_4_ | M-H | 0.99 | 1.20E-08 | 2.06E-06 | -0.65 | 4.79E-05 | 0.00296 |
| 228.0944 | 8.38 | Deoxycytidine | C_9_H_13_N_3_O_4_ | M+H | -0.36 | 6.35E-06 | 0.00016 | 0.57 | 5.68E-06 | 0.00120 |
| 155.0186 | 6.62 | Orotate | C_5_H_4_N_2_O_4_ | M-H | -1.44 | 0.00158 | 0.00721 | 1.14 | 0.00734 | 0.04303 |
| 244.0635 | 4.37 | Pseudouridine | C_9_H_12_N_2_O_6_ | M-H | -1.21 | 0.00017 | 0.00140 | 1.72 | 0.00170 | 0.02328 |
| 241.0923 | 11.82 | Thymidine | C_10_H_14_N_2_O_5_ | M+Hac-H | 0.34 | 0.00888 | 0.02756 | -0.37 | 0.00496 | 0.04298 |
| 111.0183 | 6.94 | Uracil | C_4_H_4_N_2_O_2_ | M-H_2_O-H | 0.23 | 0.00050 | 0.00301 | -0.30 | 1.20E-05 | 0.00143 |
| 112.0497 | 8.39 | Cytosine | C_4_H_5_N_3_O | M+H | -0.40 | 0.00000 | 0.00010 | 0.24 | 0.00334 | 0.03256 |
| 220.1188 | 8.75 | Pantothenic acid | C_9_H_17_NO_5_ | M+H | 0.36 | 0.02282 | 0.04788 | -0.41 | 0.01225 | 0.04753 |
| 401.3421 | 3.29 | 7-Oxocholesterol | C_27_H_44_O_2_ | M+H | -1.12 | 0.00106 | 0.00577 | 1.36 | 0.00260 | 0.02948 |
| 124.0059 | 8.35 | Taurine | C_2_H_7_NO_3_S | M-H | -0.76 | 0.00059 | 0.00336 | 0.99 | 3.77E-05 | 0.00271 |
| 191.0187 | 9.15 | Citrate | C_6_H_8_O_7_ | M-H | 0.55 | 1.23E-07 | 5.18E-06 | -0.20 | 0.00370 | 0.03532 |
| 191.0188 | 10.07 | Iso-Citric acid | C_6_H_8_O_7_ | M-H | 0.40 | 2.33E-07 | 8.44E-06 | -0.28 | 2.08E-05 | 0.00207 |
| 272.0938 | 10.07 | 5-Hydroxymethylcytidine | C_10_H_15_N_3_O_6_ | M-H | 0.67 | 8.27E-07 | 2.30E-05 | -0.59 | 8.39E-06 | 0.00118 |

**Notes:** Raw *P* values were adjusted using the Benjamini–Hochberg FDR method within each pairwise comparison. The table lists metabolites that met the FDR < 0.05 criterion in the corresponding comparisons and were used for downstream overlap analysis.

**Supplementary Figures**

**Figure S1. Intermittent fasting partially reshapes gut microbial diversity and restores *Akkermansia* abundance in DCM mice, related to Figure 2. A-C.** Alpha-diversity analyses of fecal microbiota among CON, DCM, and DCM-IF groups, assessed by Shannon index (A), Simpson index (B), and Chao1 index (C) (n = 6 per group). **D.** Relative abundance of dominant bacterial genera across groups, shown as stacked area plots (top genera, n = 6 per group). **E.** Relative abundance of *Akkermansia* at the species level, including *A. muciniphila* and other *Akkermansia* taxa, across groups (n = 6 per group). **F.** Cladogram showing taxonomic differences among CON, DCM, and DCM-IF groups based on LEfSe analysis. Differentially enriched taxa are highlighted according to the group in which they are most abundant. Data are presented as box-and-whisker plots (median, interquartile range, and min–max) in (A–C), and as relative abundance plots in (D–E). Statistical significance was assessed as indicated in the panels. **P* < 0.05.

**Figure S2. Glycaemic status and echocardiographic structural parameters in microbiota- and A. muciniphila–based intervention experiments, related to Figure 3. A.** Blood glucose levels in CON, DCM, DCM-IF, DCM-IF+ABX, and DCM+FMT groups (n = 6 per group). **B-C.** Left ventricular internal diameter at end-systole (LVIDs, B) and end-diastole (LVIDd, C) measured by echocardiography in CON, DCM, DCM-IF, DCM-IF+ABX, and DCM+FMT groups (n = 6 per group). **D-F.** Alpha-diversity analyses of fecal microbiota in CON, DCM, DCM-IF, DCM-IF+ABX, and DCM+FMT mice, assessed by Shannon index (D), Simpson index (E), and Chao1 index (F) (n = 6 per group). **G.** Principal coordinates analysis (PCoA) based on Bray–Curtis distances showing fecal microbial community structure across CON, DCM, DCM-IF, DCM-IF+ABX, and DCM+FMT groups. **J.** Blood glucose levels in CON, DCM, and DCM+AKK groups (n = 6 per group). **K-L.** Echocardiographic assessment of LVIDs (E) and LVIDd (F) in CON, DCM, and DCM+AKK groups (n = 6 per group). Data are presented as mean ± SD with individual animals shown as dots, or as box-and-whisker plots (median, interquartile range, and min–max) where indicated. Statistical significance was assessed by one-way ANOVA followed by Dunnett’s multiple-comparisons test, or by nonparametric tests where appropriate. For G, group differences were assessed by PERMANOVA based on Bray–Curtis distances. **P* < 0.05, ***P* < 0.01, ****P* < 0.001.

**Figure S3. Additional metabolomic analyses, 1-methyl-L-histidine quantification, and in vitro growth assessment related to Figure 4. A.** PCA score plots of untargeted metabolomics acquired in negative-ion mode (ESI−) in serum (left) and heart tissue (right) among CON, DCM, and DCM-IF groups (n = 8 per group). **B-D.** Levels of 1-methyl-L-histidine in feces (B), serum (C), and heart tissue (D) among CON, DCM, DCM-IF, DCM-IF+ABX, and DCM+FMT groups (n = 6 per group). FMT indicates mice receiving fecal microbiota transplantation from DCM-IF donors. **E.** In vitro single-microbial transformation assay showing *A. muciniphila* co-cultured with 100 μM L-histidine or 100 μM L-anserine for 24 h, together with OD600 measurements of bacterial growth. **F.** In vitro mixed-microbial transformation assay using fecal microbial communities from CON, DCM, and DCM+AKK mice, followed by co-culture with 100 μM L-histidine or 100 μM L-anserine for 24 h, together with OD600 measurements of microbial growth. Data are presented as violin plots with median and quartile lines in (B–D), and as mean ± SD with individual replicates shown as dots in the OD600 bar plots in (E) and (F). Statistical significance was assessed by one-way ANOVA followed by Dunnett’s multiple-comparisons test, or by nonparametric tests where appropriate. **P* < 0.05, ***P* < 0.01, ****P* < 0.001.

**Figure S4. Additional validation of 1-methyl-L-histidine biology and cardiac structural parameters in the 1-methyl-L-histidine intervention experiment, related to Figure 5. A.** Fecal levels of 1-methyl-L-histidine in CON, DCM, and DCM+His groups (n = 6 per group). **B.** Blood glucose levels in CON, DCM, and DCM+His groups (n = 6 per group). **C-D.** Echocardiographic measurements of left ventricular internal diameter at end-systole (LVIDs, C) and end-diastole (LVIDd, D) in CON, DCM, and DCM+His groups (n = 6 per group). Data are presented as violin plots with median and quartile lines in (A), and as mean ± SD with individual animals shown as dots in (B–D). Statistical significance was assessed by one-way ANOVA followed by Dunnett’s multiple-comparisons test, or by nonparametric tests where appropriate. **P* < 0.05, ****P* < 0.001.

**Figure S5. Associations between cardiac 1-methyl-L-histidine levels and lipid peroxidation susceptibility indices, and additional validation of oxidative injury across intervention paradigms, related to Figure 7. A-B.** Pearson correlation analyses showing the relationships between cardiac 1-methyl-L-histidine levels and lipid unsaturation metrics, including unsaturation index (UI, A) and peroxidizability index (PI, B). Pearson correlation coefficients (R) and two-tailed P values are indicated. **C.** Representative immunohistochemical staining of 4-hydroxynonenal (4-HNE) and 8-hydroxy-2’-deoxyguanosine (8-OHdG) in myocardial sections from CON, DCM, and DCM-IF groups, together with quantification of 4-HNE-positive and 8-OHdG-positive areas (n = 3 per group). **D.** Representative 4-HNE and 8-OHdG staining in myocardial sections from CON, DCM, DCM-IF, DCM-IF+ABX, and DCM+FMT groups, together with quantification of 4-HNE-positive and 8-OHdG-positive areas (n = 3 per group). **E.** Representative 4-HNE and 8-OHdG staining in myocardial sections from CON, DCM, and DCM+AKK groups, together with quantification of 4-HNE-positive and 8-OHdG-positive areas (n = 3 per group). Data are presented as mean ± SD. One-way ANOVA followed by Dunnett’s multiple comparisons test was used for comparisons among multiple groups. **P* < 0.05, ***P* < 0.01, ****P* < 0.001.

**Figure S6. Additional immunoblot validation with expanded biological replicates, related to Figures 1, 3, and 5. A.** Additional immunoblot images corresponding to the IF intervention experiment in CON, DCM, and DCM-IF mice. **B.** Additional immunoblot images corresponding to the ABX/FMT experiment in DCM, DCM-IF, DCM-IF+ABX, and DCM+FMT mice. **C.** Additional immunoblot images corresponding to the *A. muciniphila* supplementation experiment in DCM and DCM+AKK mice. **D.** Additional immunoblot images corresponding to the 1-methyl-L-histidine supplementation experiment in DCM and DCM+His mice. Immunoblots show α-SMA, BAX, BCL-2, Caspase 3, Cleaved caspase 3, and GAPDH or β-actin as indicated.

These images correspond to the remaining three biological replicates per group that were additionally analyzed during revision. Quantification in the revised main figures was based on six biological replicates per group pooled from the original and additional immunoblot analyses.
